# An Ethnobotanical Study of Medicinal Plants in Mersin (Turkey)

**DOI:** 10.3389/fphar.2021.664500

**Published:** 2021-07-07

**Authors:** Gizem Emre, Ahmet Dogan, Mehmet Zeki Haznedaroglu, Ismail Senkardes, Mahmut Ulger, Aysen Satiroglu, Berivan Can Emmez, Osman Tugay

**Affiliations:** ^1^ Department of Pharmaceutical Botany, Faculty of Pharmacy, Marmara University, Basibuyuk-Istanbul, Turkey; ^2^ Department of Pharmaceutical Botany, Faculty of Pharmacy, Izmir Katip Celebi University, Cigli-Izmir, Turkey; ^3^ Department of Pharmaceutical Microbiology, Faculty of Pharmacy, Mersin University, Yenisehir-Mersin, Turkey; ^4^ Department of Sociology, Faculty of Letters, Istanbul University, Fatih-Istanbul, Turkey; ^5^ Department of Turkish Language and Literature, Faculty of Humanities and Social Sciences, Adana Alparslan Turkes University, Adana, Turkey; ^6^ Department of Pharmaceutical Botany, Faculty of Pharmacy, Selcuk University, Selcuklu-Konya, Turkey

**Keywords:** Ethnobotany, folk medicinal plants, traditional knowledge, Mersin, Turkey

## Abstract

This comprehensive ethnobotanical study carried out in Mersin province, which is located in the southern part of Anatolia, east of the Mediterranean Sea, compiles details on plants used in folk medicine and ethnopharmacological information obtained through face-to-face interviews. The aim was to collect and identify plants used for therapeutic purposes by local people and to record information on traditional herbal medicine. Plant specimens were collected in numerous excursions. Additionally, informant consensus factor and use value (UV) were calculated for information gathered. This study identifies 93 plant taxa belonging to 43 families and records their usage in folk medicine; 83 taxa are wild and the remaining 10 are cultivated. The most commonly used plants belong to Lamiaceae, representing 15.0% of the total, while the Rosaceae, Malvaceae, Hypericaceae, Asteraceae and Cupressaceae families each represented another 5.4%. As a result of this investigation, we determine 189 medicinal usages of 93 taxa. The UV values indicate that the most important medicine plants are *Hypericum perforatum* (0.80), *Cedrus libani* (0.78), *Quercus coccifera* (0.77), *Arum dioscoridis* (0.76) and *Juniperus drupaceae* (0.74). We observed that most of the drugs are prepared using the infusion method (27.6%). As a conclusion, the study finds that traditional folk medicine usage is still common, especially among the rural population of Mersin.

## Introduction

The Mediterranean area, which possesses a unique ecology with various natural features, has been inhabited for millennia and is strongly influenced by human–nature relationships (Scherrer et al., 2005). The tradition of using wild plants for medicinal reasons continues in today’s small rural communities, especially among societies that maintain the cultural bridge between past and present. While the recently developed fast communication technologies connect people in seconds and spread data across vast distances, traditional knowledge still holds importance in daily life. Over the past few decades, efforts to preserve traditional knowledge have escalated around the world, especially in Europe and Mediterranean countries (Varga et al., 2019).

Besides being home to many plants in floristic terms, Turkey is rich in traditional herbal medicine, in addition to its cultural, historical and geographical heritage (Bulut et al., 2013). Ethnobotanical studies show that traditional knowledge of medicinal plants still exists in the Mediterranean Region, especially among elderly (Agelet, et al., 2003). Many scientists have focused on such studies and governmental foundations have increased financial support of this kind of research. The Turkish Ministry of Agriculture and Forestry has organized studies across the country in the scope of the “Recording of Traditional Knowledge Based on Biological Diversity Project.”

The Taurus Mountains are one of the highlights of the Mediterranean Region with a rich plant diversity (Everest et al., 2005). Mersin has previously been the subject of this kind of scientific research, such as a study on herbal drugs on herbal markets in Mersin, which was conducted throughout the entire province (Everest et al., 2005). Thorough documentation of the traditional use of medicinal plants across the entirety of Mersin province is not presently available. Three districts (Sargın 2015; Sargın et al., 2015; Sargin and Büyükcengiz, 2019) and some specific areas of the province have been investigated from an ethnobotanical perspective. Another study investigates a small section of the region (Akaydın et al., 2013); however, as one of the largest cities in Turkey, Mersin needs further investigation from an ethnobotanical perspective.

We aim to record the traditional usage of medicinal plants by conducting an ethnobiological study in Mersin that covers various different altitudes and areas representing all ten of its districts.

To this end, we compare the gathered ethnomedicinal data with previous findings from the Balkan and Mediterranean regions. We highlight new plants and usages from the region for future phytochemical and phytopharmacological studies. With further cultivation studies, these findings may demonstrate the potential for economic development for the benefit of local communities.

Hypothesis of this study tests;

a Traditional knowledge is still being used in villages far from the city and main settlement centers,
b Plants are still being used in the more isolated villages.

## Materials and Methods

**FIGURE 1:**
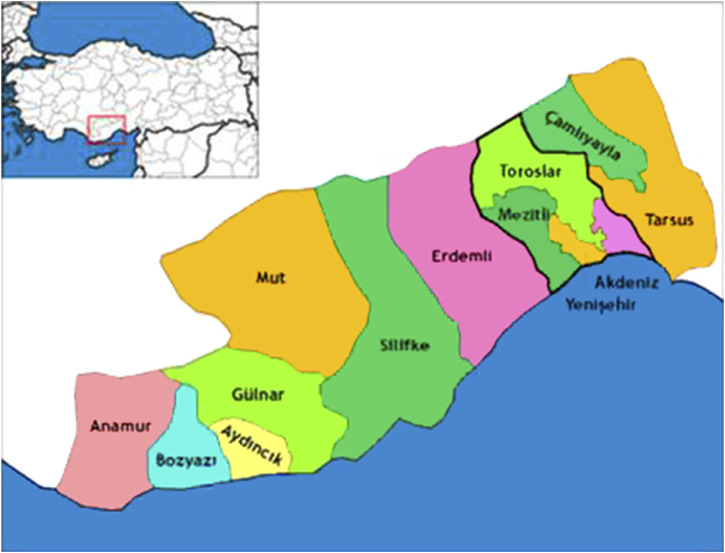
Map of mersin (https://tr.wikipedia.org).

**FIGURE 2:**
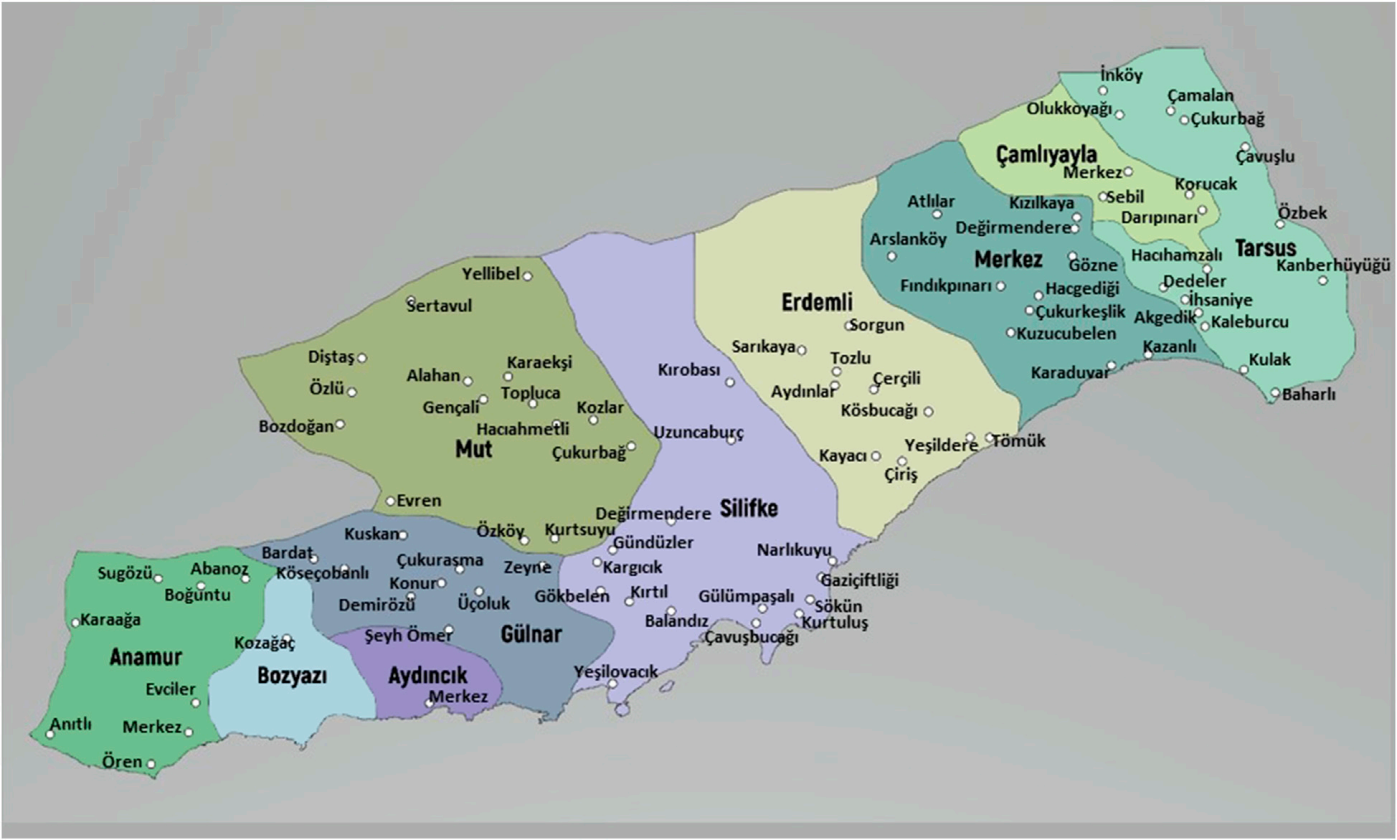
Map of visited villages of study area.

Mersin is a province in southwestern Anatolia, located at a latitude of 36^°^ 37′ north and a longitude of 33^°^ 35’ east; covering a 15.853 km^2^ area with a population of 1,814,468 (http://www.tuik.gov.tr) (Figure 1). The majority of the acreage is mountainous (87%) and forestland is 54%. There are ten districts: Anamur, Aydincik, Bozyazi, Camliyayla, Erdemli, Gulnar, Merkez, Silifke, Mut, and Tarsus. This ethnobotanical survey includes 91 villages located in all ten districts of Mersin (Figure 2).

The territory of the province consists primarily of the high, rugged, rocky Western and Central Taurus Mountains. The highest point in Mersin is Mount Medetsiz (3,585 m) in the Bolkar Mountains. The altitude decreases from northwest toward the south. Kumpet Mountain (2,473 m), Elma Mountain (2,160 m), Alamusa Mountain (2,013 m), Big Egri Mountain (2,025 m), Kızıl Mountain (2,260 m), Naldoken Mountain (1,754 m), and Kabakli Mountain (1,675 m) are the topographic heights from the Bolkar Mountains in the west.

Karaziyaret Mountain, Tol Mountain, Sunturas Mountain, Balkalesi, Ayvagedigi Mountain, Makam Mountain and Kaskaya Mountain are other important elevations heading toward the south. Mersin is connected to Central Anatolia through Gulek Pass (1,050 m) from the northeast and Sertavul Pass (1,610 m) from northwest.

**FIGURE 3:**
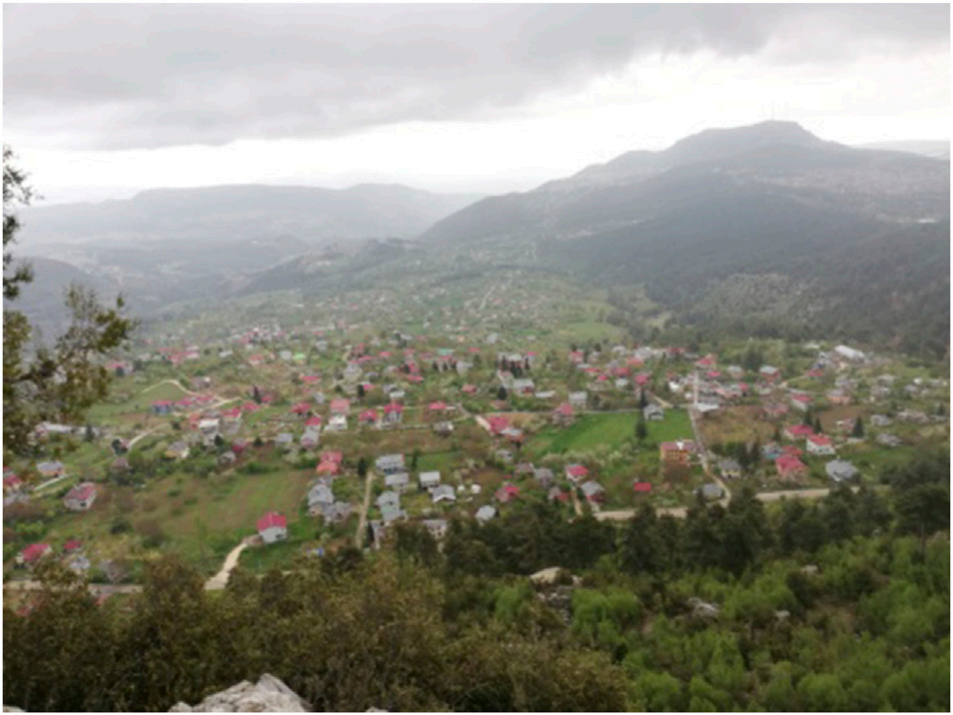
View of Camliyayla highland.

Rivers, streams, atmospheric conditions and the tectonic faults in the region give rise to various plains in the upper reaches of the Taurus Mountains, with altitudes ranging from 700 to 1,500 m. Major plateau areas of Mersin include the highlands of Aslankoy, Gozne, Findikpinari, Sogucak, Bekiralani, Mihrican, Ayvagedigi and Guzelyayla, Camlıyayla, Gulek and Sebil, Sorgun, Kucuk Sorgun, Toros, Kucukfındıklı and Guzeloluk, Balandiz, Uzuncaburc, Gokbelen and Kirobasi, Abanoz, Kas and Besoluk, Bozyazi, Elmagozu and Kozagac, Bardat, Tersakan and Bolyaran, Kozlar, Civi, Dagpazari, Sogutozu and Sertavul (Figure 3). The province is not rich in terms of rivers. The most important rivers are the Goksu and Berdan streams.

The climate is Mediterranean with an annual mean temperature of 22°C and a mean rainfall of 1,096 mm per year (Meteroloji Genel Müdürlüğü, 2020).

The primary sources of income in Mersin are industry (40%), agriculture (30%), and trade/business sector (10%).

The main crops of Mersin are wheat, barley and cotton. Mersin plays an important role in greenhouse cultivation of various agricultural products, of which banana production in Anamur is one of the most famous. *Citrus* trees, tropical fruits and vegetables are also commonly cultivated.

The vegetation of Mersin district presented here is based on the authors’ own observations and field records. Mersin, which is generally covered with maquis or forest vegetation, contains Mediterranean elements. In areas with maquis, plants such as *Ceratonia siliqua* L.*, Cistus creticus* L., *Laurus nobilis* L., *Myrtus communis* L.*, Nerium oleander* L.*, Paliurus spina-christi* P. Mill., *Phillyrea latifolia* L.*,* and *Quercus coccifera* L. are widespread. Tree species such as *Pinus nigra* J.F.Arnold subsp. *pallasiana* (Lamb.) Holmboe, *Cedrus libani* A. Rich. var. *libani*, *Abies cilicica* (Antoine and Kotschy) Carriere subsp. *cilicica*, *Juniperus excelsa* M. Bieb. subsp. *excelsa, J. foetidissima* Willd.*, J. oxycedrus* L. subsp. *oxycedrus,* are observed in high altitudes (above 900 m). Lowland forest areas usually consist of *Pinus brutia* Ten. (Davis, 1965; Davis et al., 1988; Güner et al., 2000).

**FIGURE 4:**
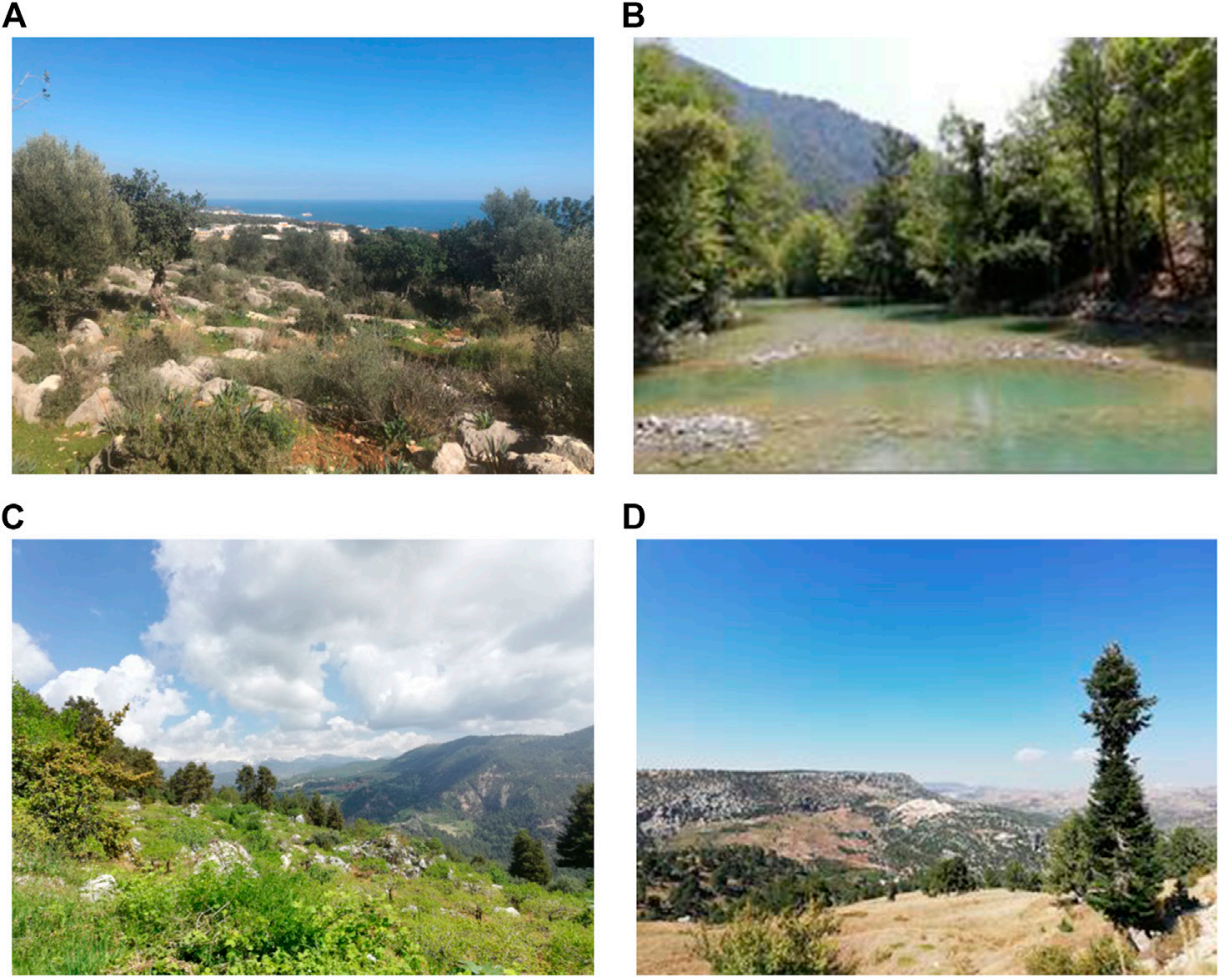
**(A‐D)** Some scenery of Mersin.

Mersin province also has significant dune and halophyte vegetation, including taxa such as *Cyperus capitatus* Vand., *Eryngium maritimum* L., *Euphorbia paralias* L., *Pancratium maritimum* L., *Halimione portulacoides* (L.) Aellen, *Juncus acutus* L. subsp. *acutus*, *J. maritimus* Lam., *Limonium virgatum* (Willd.) Fourr. and *Tamarix smyrnensis* Bunge (Davis, 1965; Davis et al., 1988; Güner et al., 2000) (Figures 4A–D).

**FIGURE 5:**
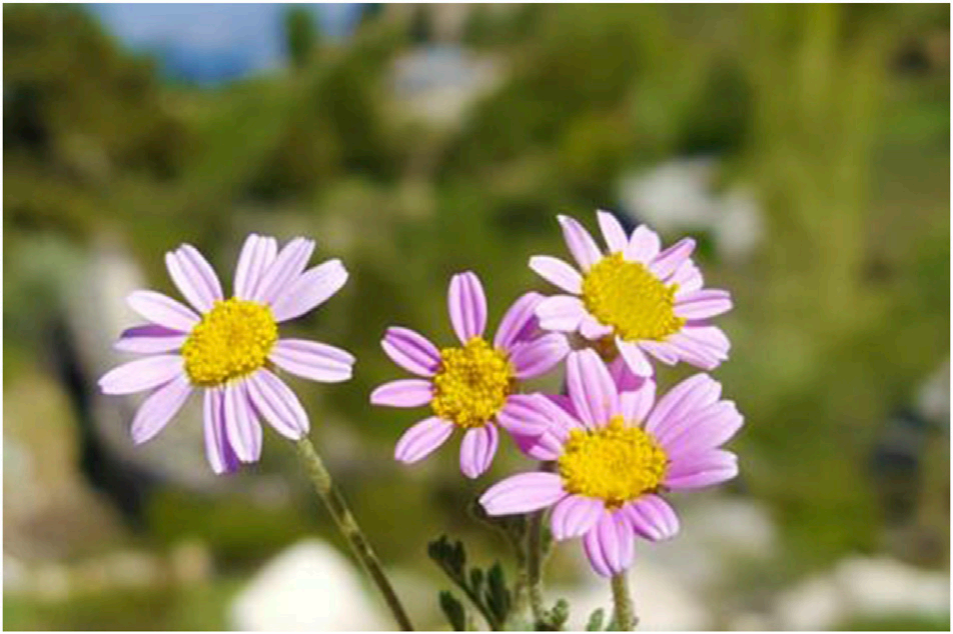
Habit view of endemic *Anthemis rosea* subsp. *carnea*.

**FIGURE 6:**
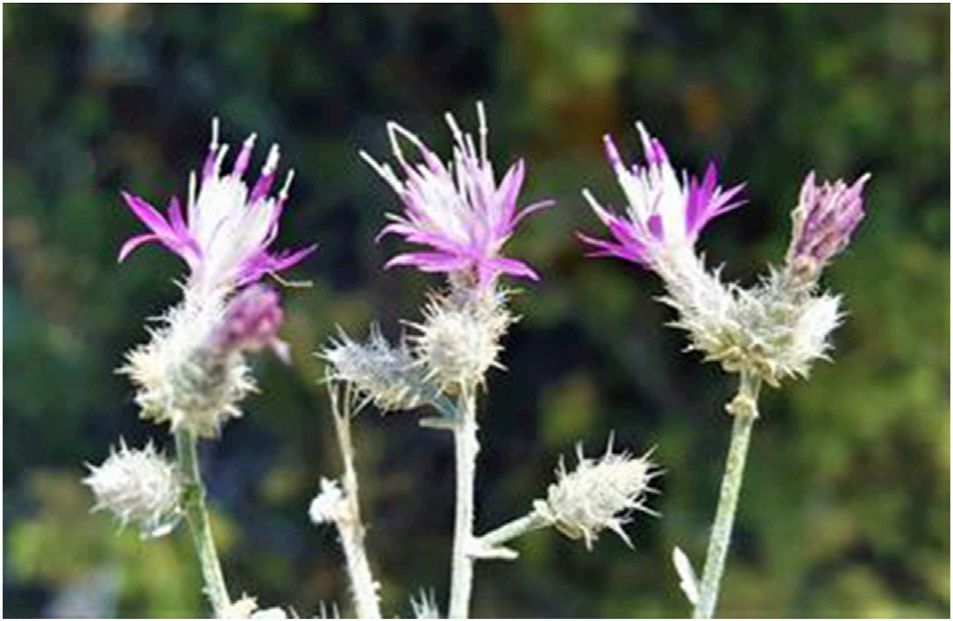
Habit view of endemic *Centaurea pinetorum*.

Some plants of Mersin are endemic to Turkey; such as *Alkanna hispida* Hub.-Mor., *Anthemis rosea* Sm. subsp. *carnea* (Boiss.) Grierson (Figure 5), *Astragalus schottianus* Boiss., *Centaurea pinetorum* Hub.-Mor. (Figure 6), *Colchicum balansae* Planch., *Crocus boissieri* Maw, *Delphinium dasystachyon* Boiss. and Balansa, *Eryngium polycephalum* Hausskn. ex H. Wolff, *Ferulago pauciradiata* Boiss. and Heldr., *Lamium eriocephalum* Benth., *Ophrys cilicica* Schltr., *Origanum boissieri* Ietsw., *Papaver pilosum* Sibth. and Sm. subsp. *glabrisepalum* Kadereit, *Pimpinella isaurica* V.A.Matthews subsp. *isaurica*, *Salvia heldreichiana* Boiss. ex Benth., and *Sideritis cilicica* Boiss. and Balansa (Davis, 1965; Davis et al., 1988; Güner et al., 2000).

### Demographic Characteristics of Respondents

As mentioned above, Mersin is one of the most populous provinces of Turkey with a population density of 114.45/km^2^. Due to the migration mobility in the region, 55,779 people moved into and 61,917 people left the city center between 2017–2018. Regarding the population growth rate, there was a notable population increase in districts close to the city center. The number of men and women living in the province is almost equal, more than half of the population are under the age of 35, 38% are 35–64 and 9% of the population are over 65. The literacy rate is 97.72 (TUIK, 2020).

The villages of Mersin province have different characteristics depending on local geographical features, such as whether they are located at high or low (near the coast) altitudes, or are near to or far from the city. There are also migrant villages and a few semi-nomadic families living in the highlands. Most of the villages in Mersin are Yoruk, alongside villages consisting of Tahtacı, Cretan and Circassian peoples. As all of the participants spoke Turkish (some elderly participants could speak Cretan and Circassian languages in addition to Turkish), we did not experience language or communication problems. Most of the remaining population of these villages is elderly. Although many of them were literate, most were at the level of primary school education.

### Data Collection

This study was conducted following the guidelines for best practices in ethnopharmacological research (Heinrich et al., 2018). Ethnobotanical data were collected in face-to-face interviews (Appendix 1) conducted in Turkish with inhabitants of Mersin on several trips to the province between 2018 and 2019. Field work was carried out over a total of 71 days. Plant vouchers were collected in collaboration with the informants. We adhered to The International Society of Ethnobiology Code of Ethics in interviews (International Society of Ethnobiology Code of Ethics with 2008 additions http://ethnobiology.net/code-of-ethics/).

A total of 338 interviews were performed. Of the participants, 247 were male and 91 were female.

The informants’ occupations were farmers, housewives, shepherds, mukhtar (village headmen), labourers (forestry workers) and cafe owners. Interviews were performed in various settings, such as coffee houses, gardens, houses and fields. Experienced adults, patients and local healers were the main source of information about local names, part(s) of plants used, ailments treated, therapeutic effects, methods of preparation and methods of administration. Interviews also covered adverse effects of folk medicines. Although the primary focus of our study was to collect information on the folkloric use of medicinal plants, animal-based remedies were also discussed and recorded.

Collected plants were identified according to “The Flora of Turkey and East Aegean Islands” (Davis, 1965; Davis et al., 1988; Guner et al., 2000) and “Illustrated Flora of Turkey Vol 2” (Güner et al., 2018). Voucher specimens were deposited at the Herbarium of the Faculty of Pharmacy at Marmara University (MARE) and Herbarium of Konya at Selçuk University (KNYA).

### Data Analyses

Informant consensus factor (Trotter and Logan, 1986; Heinrich et al., 1988) was calculated according to the following formula: *FIC =Nur–Nt/Nur-1*, where *Nur* refers to the number of citations used in each category and *Nt* to the number of species used. This method demonstrates the homogeneity of the information: if plants are chosen randomly or if informants do not contribute information about their use, FIC values will be close to zero. If there is a well-defined selection criterion in the community and/or if information is given between the informants’ values, the value will be close to one (Afifi and Abu-Irmaileh, 2000; Abu-Irmaileh and Afifi, 2003). Medicinal plants with higher FIC values are considered to be more likely to be effective in treating a certain disease (Teklehaymanot and Giday, 2007).

A quantitative method called “use value” (UV_s_), calculated according to the formula UVs (medicinal use value) parameter using the Phillips and Gentry, 1993 formula as modified and used by Thomas et al., 2009:

$$UVs=\sum ni=1U_{\text{is}}/ns$$

in which UV_s_ is the use value of a given species s, *U*_is_ is the number of uses of species s listed by the informant i, and ns is the total number of informants.

We used the most common method of dendogram clustering to demonstrate the relationship of the taxa and traditional usages in ten different districts of Mersin. The Unweighted Pair Group Method with Arithmetic Mean (UPGMA) was used for statistical analysis with v2. (Sokal and Michener, 1958; Bailey, 1994).

The proportion and pairwise-proportion (with Holm adjustment) tests were used to compare the true (population) proportions. These tests were performed in R and the significance level was fixed at 0.05.

## Results

### Demographic Features of the Informants

Details on the demographic characteristics of the participants were asked in face-to-face interviews. Among 338 participants, 16 were 19–35 years of age, 40 were 36–49, 194 were 50–70 and 88 were over the age of 70. The majority of the respondents were male (247) and 91 were female.

The age of the informants ranged between 19 and 91 years old with a mean age of 68 years.

**FIGURE 7:**
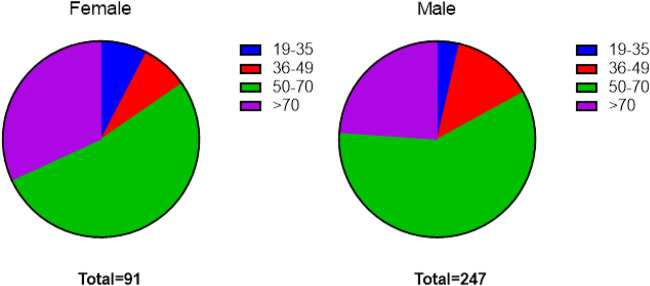
Age groups of participants.

**FIGURE 8:**
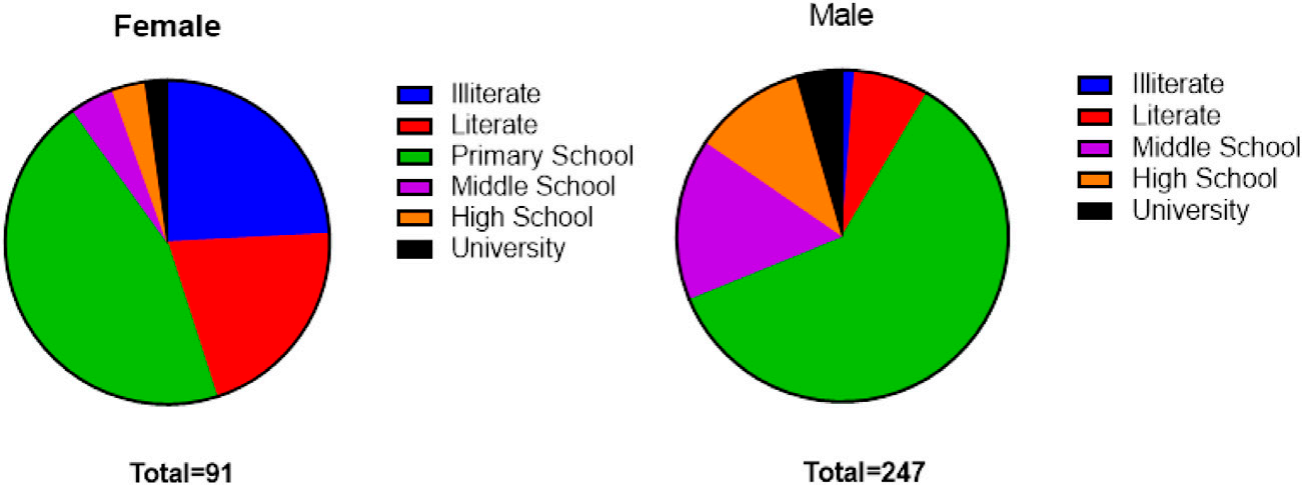
**(A–B)** Educational status of participants.

Among all the participants; 25 were illiterate (7%), 37 were literate (11%), 190 had graduated from elementary school (57%), 43 from middle school (12%), 30 from high school (9%) and 13 from university (4%) (Figures 7A,B, 8A,B).

The occupational groups of the participants consist of farming, animal husbandry, beekeeping, shepherding, retired, tradesmen and housewives. We gained access to four local healers, who can be regarded as practitioners of traditional medicine, for this study.

It should be noted that the reason women informants constituted only one third of the total number is that the study started mostly in the coffeehouses, which were generally in the center of the villages and in Turkey are frequented only by men.

### Medicinal Plants and Related Knowledge

The plants used for medicinal treatment of human beings in Mersin are listed in Table 1, while Table 2 shows the plants that see veterinary use. Both are arranged alphabetically by botanical name and include relevant information. Taxonomic changes according to The Plant List (The Plant List, 2013) are shown in parentheses with scientific names in Table 1. In total, 324 plant specimens were collected in the research area during the study period. Among these, 93 medicinal plants belonging to 43 families were identified; of these 83 taxa were wild and 10 were cultivated. The most commonly used medicinal plants were in the Lamiaceae (14 taxa), Rosaceae (5 taxa), Malvaceae (5 taxa), Hypericaceae (5 taxa), Asteraceae (5 taxa) and Cupressaceae (5 taxa) families.

**TABLE 1:** Folk medicinal plants of Mersin (Turkey).

Botanical name, family and specimen number	Local name	Plant part used	Ailments treated/Therapeutic effect	Preparation	Administration	UV	Ethnobotanical records from Turkey	Ethnobotanical records from mediterranean region
***Abies cilicica*** (Ant. et Kotschy) Carr.	Iladin	Resin	Wounds	Crushed with olive oil	Ext	0.44	(6, 9, 10, 11)^b^	—
Pinaceae, MARE 21101	—	—	—	—	—	—	—	—
***Achillea*** sp.
Asteraceae, MARE 21004	Kirkbas	Capitulum	Abdominal pain	Infusion	Int	0.34	—	—
***Alcea pallida*** (Willd.) Waldst. et Kit.	Aysefatma, Ayigulu, Esefatma cicegi, Hasbi cicegi	Flowers	Expectorant, cough	Decoction	Int	0.41	Cough (3)	—
Malvaceae, MARE 20130, 21063							(3)^b^	
***Alcea rosea*** L.^a^	Aysefatma, Gulhatmi	Flowers	Cough	Decoction	Int	0.15	—	(31, 54)^b^
Malvaceae, MARE 20173								
***Arbutus*** x ***andrachnoides*** Link	Sandal	Fruits	**Abdominal pain**	—	Int	0.24	—	—
Ericaceae, MARE 20175, 21068, 21157								
***Arum dioscoridis*** Sm. Araceae, MARE 20056, 20057	Agi otu, Agu, Elkabartan, Tirsin, Yilanbicagi, Yilanekmegi	Fruits leaves	Bee bite	Crushed	Ext	0.76	Hemorrhoids (6, 8, 9)	—
			Abdominal pain	—	Int		(9)^b^	
			Hemorrhoids	—	2 pieces in a day for 2 months, int		—	
			Cough	Heated then mixed with olive oil	Ext		—	
			Sore throat	Heated then mixed with olive oil	Ext		—	
***Asparagus acutifolius*** L.	Kuskonmaz	Aerial parts	Urinary system diseases	Infusion	Int	0.13	(5, 6, 7, 8, 11)^b^	(15, 17, 22, 35, 41, 44, 49, 51, 54)^b^
Asparagaceae, MARE 20150, 21162, 21637								
***Asphodeline lutea*** (L.) Rchb.	Kecicirisi	Leaves	**Burns**	—	Wrapped in a cloth, ext	0.11	—	—
Xanthorrhoeaceae, MARE 21645								
***Asphodelus aestivus*** Brot.Xanthorrhoeaceae, MARE 21527, 21680	Ciris	Tuber	Wounds	Crushed then added flour	Wrapped in a cloth for 3 days, ext	0.20	Wounds (7)	Wounds (15)
							(5, 7, 8)^b^	(15)^b^
***Asphodelus fistulous*** L.	Ciris	Tuber	**Wounds**	Crushed then mixed with olive oil	Wrapped in a cloth, ext	0.17	—	—
Xanthorrhoeaceae, MARE 20061								
***Astragalus*** sp.	Geven, Keven	Resin	Herniated disc	—	Wrapped in a cloth for 3 days, ext	0.24	—	—
Fabaceae, MARE 21046, 21054								
***Berberis crataegina.*** DC.	Karamik	Leaves	Diabetes, diarrhea	—	Int	0.17	Diabetes (4)	—
		Stem bark	Diabetes	Decoction	Int		(2, 3, 4, 5)^b^	
Berberidaceae, MARE 21007, 21931		Fruits	Stye	—	Ext		—	
							—	
***Cedrus libani*** A. Rich. Pinaceae, MARE 20084, 20192, 21104, 21504	Katran, Sedir	Resin	Stomachache	—	Int	0.78	Wounds (3, 10)	—
			Eczema, Warts, wounds	—	Ext		(2, 3, 6, 9, 10)^b^	
***Ceratonia siliqua*** L. Fabaceae, MARE 20170, 21066	Hunnap, Kara armut	Fruits	Cough, Anemia	Boiled (called pekmez)	Int	0.61	Anemia (7,8,9,10)	(17, 22, 28, 35, 36)^b^
			Appetite	Boiled	Ext		Appetizer (9)	
			Abscess	—	—		Cough (1)	
							(1, 2, 5, 6, 7, 8, 9, 10)^b^	
***Ceterach officinarum*** DC.	Altin otu, Yosun cayi	Aerial parts	Stomachache	Infusion	Int	0.10	Stomachache (7) (3, 7, 8, 11)^b^	(12, 17, 22, 26, 27, 28, 35, 39, 44, 45, 48, 51)^b^
Aspleniaceae, MARE 21529 [*Ceterach officinarum* Willd.]								
***Cichorium intybus*** L. Asteraceae, MARE 21049, 21060	—	Aerial parts	Stomachache	Infusion	Int	0.09	Stomachache (8), (4, 8)^b^	(13, 17, 25, 29, 31, 35, 36, 37, 38, 41, 44, 45, 49, 51, 55)^b^
***Cistus creticus*** L. Cistaceae, MARE 21119	Karahan	Aerial parts	Antifungal	Decoction	Ext	0.08	(3, 6, 7, 8)^b^	(15)^b^
***Cistus salviifolius*** L. Cistaceae, MARE 21013	Karahan	Aerial parts	**Antifungal**	Decoction	Ext	0.08	(7, 8)^b^	—
***Crataegus monogyna*** Jacq. subsp. ***monogyna*** Rosaceae, MARE 21001, 21914, 21916 (*Crataegus monogyna* Jacq.)	Alic	Aerial parts	Stomachache, cardiovascular system diseases	Infusion	Int	0.31	Cardiovascular system diseases (9), (3, 4, 6, 8, 9)^b^	Cardiovascular system diseases (15, 17, 19, 24, 31), Stomachache (35), (12, 13, 16, 17, 19, 24, 25, 27, 28, 31, 32, 33, 34, 35, 36, 37, 38, 39, 43, 47, 51, 53, 54, 55)^b^
***Cupressus sempervirens*** L.^a^(Cupressaceae, MARE 21059, 21303)	Selvi	Cones	Cough	Decoction	Int	0.08	(2, 5)^b^	(15, 17, 29, 42, 50)^b^
***Cydonia oblonga*** Mill. Rosaceae, MARE 21084, 21678	Ayva	Leaves	Diarrhea, Stomachache Sore throat	Infusion boiled in water	Int. Wrapped in a cloth, Ext	0.64	Diarrhea (1, 3, 6, 9, 10), (1, 3, 4, 5, 6, 9, 10)^b^	Diarrhea (20, 24, 28, 31, 38, 44, 53, 54), Stomachache (31), (12, 17, 21, 28, 31, 37, 39, 40, 42, 46, 52)^b^
***Ecballium elaterium*** (L.) A. Rich. Cucurbitaceae, MARE 21114, 21663	Cirtatan, cakalkavunu	Fruits juice (fresh), fruits	Haemorrhoids sinusitis rheumatism	— — Heated in the olive oil	Ext. Intranasal drops Ext	0.39	Hemorrhoids (3.8), rheumatism (9), sinusitis (1, 3, 6, 8, 9, 10, 11), (1, 6, 8, 9, 10, 11)^b^	Sinusitis (52) (21, 40, 45, 51, 53)^b^
***Equisetum telmateia*** Ehrh. Equisetaceae, MARE 21087	Kirkkilit	Aerial parts	Diuretic	Infusion	Int	0.13	Edema (3)	Diuretic (17, 25, 28, 53) (17, 23, 24, 25, 28, 40, 50, 53)^b^
***Eriobotrya japonica*** (Thunb.) Lindl.^a^ Rosaceae, MARE 21309	Yenidunya, Malta erigi	Flowers	Sore throat,cough	Infusion	Int	0.32	Cough (1), (1, 9, 10)^b^	(17, 22, 40, 52)^b^
***Eucalyptus camaldulensis*** Dehnh. Myrtaceae, MARE 21105, 21174, 21891	Kaliptos, Okaliptus, Sitma agaci	Leaves	Cough	Infusion	Int	0.08	(1, 6, 8, 9, 10)^b^	Cough (21, 25), (15, 17, 25)^b^
***Euphorbia helioscopia*** L. Euphorbiaceae, MARE 21300	Sutlegen	Latex	Warts	—	Ext	0.63	—	Warts (33), (27, 51)^b^
***Euphorbia kotschyana*** Fenzl Euphorbiaceae, MARE 21092	Sutlegen	Latex	Warts	—	Ext	0.63	(2)^b^	—
***Euphorbia rigida*** M. Bieb. Euphorbiaceae, MARE 21318	Sutlegen	Latex	Warts	—	Ext	0.63	Warts (3.9), (3, 6, 8, 9, 10)^b^	—
***Ficus carica*** L. subsp. ***carica*** ^a^, Moraceae, MARE 20180, 21151 [*Ficus carica* L.]	Incir	Latex	Toothache, warts	—	Ext	0.71	Toothache (6), warts (3, 9), (1, 2, 3, 4, 6, 7, 9, 10)^b^	Toothache (41), warts (13, 17, 20, 22, 24, 25, 27, 28, 29, 31, 34, 35, 36, 39, 41, 44, 50, 52), (13, 15, 17, 22, 25, 26, 27, 31, 32, 33, 35, 36, 41, 45, 48, 50, 53, 55)^b^
***Foeniculum vulgare*** Mill. Apiaceae, MARE 21021	Arapsaci, Meletura, Rezene	Aerial parts	Abdominal pain	Infusion	Int	0.41	Abdominal pain (3) (1,4,8)^b^	Abdominal pain (32, 33, 34) (13, 15, 17, 20, 21, 22, 24, 25, 27, 28, 29, 31, 32, 33, 34, 35, 36, 37, 38, 41, 42, 43, 44, 48, 49, 50, 51, 53, 54, 55)^b^
***Glaucium flavum*** Crantz Papaveraceae, MARE 21870	Gogundurme	Latex Aerial parts	**Warts wounds**	Infusion	Ext. Int	0.07	—	—
***Gundelia tournefortii*** L. var. ***tournefortii*** (Asteraceae, MARE 21917) [***Gundelia tournefortii*** L.]	Kengel	Seeds	Stomachache	Roasted, boiled with water (made coffee)	Int	0.23	(8)^b^	—
***Hedera helix*** L. Araliaceae, MARE 21058	—	Leaves	Abscess	Heated	Ext	0.11	(5)^b^	(12, 13, 17, 22, 26, 27, 28, 29, 35, 43, 48, 51, 55)^b^
***Helichrysum compactum*** Boiss. Asteraceae, MARE 20091	Altinbas, Koyungozu	Aerial parts	**Abdominal pain**	Infusion	Int	0.31	—	—
***Hypericum atomarium*** Boiss. Hypericaceae, MARE 20117	Kantaron	Aerial parts	**Wounds**	Oleate	Ext	0.44	—	—
***Hypericum lydium*** Boiss. Hypericaceae, MARE 21097	Kantaron	Aerial parts	**Wounds**	Oleate	Ext	0.32	—	—
***Hypericum montbretii*** Spach Hypericaceae, MARE 20067	Kantaron	Aerial parts	Wounds	Oleate	Ext	0.32	Wounds (7), (7)^b^	—
***Hypericum perforatum*** L. Hypericaceae, MARE 20080, 20997, 21091, 21549	Kantaron, Koromaz	Aerial parts	Stomach ailments wounds Diaper rash (in babies) Haemorrhoids Rheumatism	Oleate	Int	0.80	Stomach ailments (3)	Hemorrhoids (37, 41, 53, 54)
				Oleate	Ext		Hemorrhoids (1)	Rheumatism (25)
				Oleate	Ext		Diaper rash (1)	Stomach disorders (39, 53)
				Oleate	Ext		Rheumatism (1)	Wounds (13, 15, 25, 26, 38, 41, 46, 51, 54, 55)
				Oleate	Ext		Wounds (3,6,9,10.11), (3,4,9,10.11)^b^	(13, 15, 16, 17, 22, 23, 25, 26, 27, 28, 32, 35, 37, 38, 39, 41, 46, 47, 48, 50, 51, 53, 54, 55)^b^
***Hypericum triquetrifolium*** Turra Hypericaceae, MARE 21009, 21039, 21109, 21129, 21158, 21178	Gavursakali, Kizilcik	Aerial parts	Rheumatism	Oleate	Ext	0.27	(7,8,9,10)^b^	—
***Juglans regia*** L.^a^, Juglandaceae, MARE 20183, 21012	Ceviz	Immature fruits, young shoots	Haemorrhoids Smoking cessation	— — Infusion	Before breakfast, int. Int	0.18	Hemorrhoids (4, 10) (3, 4, 6, 7, 8, 9, 10, 11)^b^	Hemorrhoids (17, 37) (13, 16, 17, 22, 24, 27, 28, 31, 33, 34, 35, 36, 37, 38, 39, 40, 41, 44, 45, 50, 53, 54)^b^
***Juniperus drupacea*** Labill cupressaceae, MARE 20070, 20165, 21023, 21548	Andiz	Cones	Expectorant, cough, Shortness of breath	Waited in a water then boiled (called pekmez)	Before breakfast, int	0.74	Cough (2)	—
			Anthelmentic	Decoction	Before breakfast, int		Asthma (4, 6, 9, 10)	
			Rheumatism	Crushed then mixed with olive oil	Ext		Rheumatism (4), (4, 6, 9, 10, 11)^b^	
***Juniperus excelsa*** M. Bieb.	Ardic	Resin	Stomachache	—	Int	0.51	(1, 3, 6, 9, 10)^b^	Stomachache (50)
Cupressaceae, MARE 20193, 21003, 21059
***Juniperus foetidissima*** Willd	—	—	—	—	—	—	—	—
Cupressaceae, MARE 21061, 21506, 21924	Ardic, Yayli ardic	Resin	Stomachache	—	Int	0.32	(3, 6, 9, 10)^b^	—
***Juniperus oxycedrus*** L. subsp. ***oxycedrus*** Cupressaceae, MARE 20160, 21103, 21505 (*Juniperus oxycedrus* L.)	Ardic, Gilik	Immature cones	Expectorant	Crushed with honey	Int	0.71	Stomach ache (3, 10) (3,6,8,9,10.11)^b^	Rheumatism (25, 27), (15, 17, 27, 54)^b^
		Cones	Rheumatism	Decoction	First applied beeswax, ext			
		Resin	Stomachache	—	Int			
		Immature cones	Stomachache	Decoction	Int			
***Laurus nobilis*** L. Lauraceae, MARE 20169, 21070	Defne, Teynel	Leaves Fruits	Diabetes Varicose Vein Rheumatism Rheumatism	Decoction Bath Crushed Boiled	Int	0.56	Diabetes (1)	Rheumatism (43, 51) (15, 16, 20, 22, 25, 27, 28, 29, 32, 33, 35, 36, 43, 44, 45 ,51, 54, 55)^b^
					Ext			
					Ext		Rheumatism (1, 4, 6, 7, 9, 10), (1,4,5,6,7,9,10,11)^b^	
					Wrapped in a cloth, ext			
***Malva parviflora*** L. Malvaceae, MARE 20088, 21034	Ebegumeci, Gomec	Roots	**Abortive**	—	Ext	0.38	—	—
***Malva sylvestris*** L.	Ebegumeci, Gomec	Roots	Abortive	—	Ext	0.38	Abdominal pain (5)	Abdominal pain (32, 33, 44, 45)
Malvaceae, MARE 21951		Aerial parts	Abdominal pain	Maseration in water added flour	Wrapped in a cloth, ext		(1, 4, 5, 6, 7, 8, 9, 10)^b^	Abortive (12), (12, 13, 15, 16, 17, 20, 21, 22, 24, 25, 26, 27, 28, 29, 31, 33, 34, 35, 36, 38, 40, 41, 43, 44, 45, 48, 50, 51, 53)^b^
***Mentha longifolia*** (L.) Hudson subsp. ***typhoides*** (Briq.) Harley var. ***typhoides***	Yarpiz	Leaves	Diarrhea	Infusion	Int. Wrapped in a cloth, ext	0.34	Rheumatism (3)	(50)^b^
Lamiaceae, MARE 21016, 21125 [***Mentha longifolia*** subsp. ***typhoides*** (Briq.) Harley]			Rheumatism Abscess	Crushed	Wrapped in a cloth, ext			(3)^b^
***Mentha*** x ***piperita*** L.^a^	Nane	Leaves	Nausea, Abdominal pain	Infusion	Int	0.68	Abdominal pain (1)	Abdominal pain (31)
Lamiaceae, MARE 21016							Nausea (1, 9) (1, 9, 10)^b^	Nausea (43), (17, 21, 28, 31, 39, 40, 43, 53, 55)^b^
***Micromeria myrtifolia*** Boiss. et Hohen.	Topuklu cay	Aerial parts	Abdominal pain	Infusion	Int	0.34	(9, 10, 11)^b^	—
Lamiaceae, MARE 21553								
***Myrtus communis*** L. subsp. ***communis***	Hambelez, Mersin, Merta, Murt	Fruits	Diabetes	—	Eaten	0.38	Diabetes (1, 3, 6, 7, 9, 10)	(15, 25, 32, 33, 35, 53)^b^
Myrtaceae, MARE 20149, 20181, 21075, 21132 [*Myrtus communis* L.]		Leaves	Constipation	Infusion	Int		Purgative (4), (1, 2, 3, 4, 6, 7, 9, 10)^b^	
***Nerium oleander*** L. Apocynaceae, MARE 20166, 21088, 21130	Agi, Sindila, Zakkum	Flowers	Rheumatism	Oleate	Ext	0.21	(1, 2, 6, 7, 9)^b^	(13., 51)^b^
***Olea europaea*** L. var. ***europaea*** ^a^, Oleaceae, MARE 21052, 21336 [*Olea europaea* L.]	Elya, Zeytin	Fruits leaves	Constipation	Oil	Int	0.70	Mouth sore (1,9,10), (1, 2, 4, 6, 7, 9, 10)^b^	Burns (17, 22, 25, 44, 50)
			Burns	Oil	Ext			Constipation (17, 22, 25)
			Earache	Oil	Eardrops			Earache (28, 29, 52)
			Mouth sore Aphtha	Boiled in water	Gargle			(12, 14, 15, 17, 19, 20, 21, 22, 24, 25, 27, 28, 29, 32, 33, 35, 36, 38, 40, 44, 45, 48, 50, 53, 54)^b^
				Chewed	Ext			—
***Origanum majorana*** L.^a^	Mercankosk	Aerial parts	Abdominal pain	Infusion	Int	0.69	(3, 4, 9, 10, 11)^b^	Abdominal pain (34)
Lamiaceae, MARE 21551								(22, 28, 34, 43, 48, 54, 55)^b^
***Origanum syriacum*** L. var. ***bevanii*** (Holmes) Ietswaart	Kekik	Aerial parts	Abdominal pain	Decoction	Int., half glass of water	0.71	Stomachache (9, 10)	
Lamiaceae, MARE 20078, 20082, 20103, 21166, 21330 [*Origanum syriacum* subsp. *bevanii* (Holmes) Greuter et Burdet]			Stomachache	Infusion	Int		(9.10)^b^	
***Origanum vulgare*** L.	Arigani, Kekik, Merdus	Aerial parts	Abdominal pain	Decoction	Int	0.66	(11)^b^	Abdominal pain (31, 33)
Lamiaceae, MARE 21020			Stomachache, Cough	Infusion	Int			Cough (25, 33, 37, 38, 43, 46), Stomachache (24, 43), (13, 15, 16, 20, 24, 25, 26, 28, 31, 35, 37, 38, 39, 42, 43, 44, 46, 48, 50, 54, 55)^b^
***Paliurus spina-christi*** Mill.	Calti, Calti dikeni	Fruits	Cough, kidney stones	Decoction	Int	0.38	Kidney stones (7, 9)	(31, 42)^b^
Rhamnaceae, MARE 20075, 20159, 21010							(4, 5, 7, 9, 10)^b^	
***Papaver macrostomum*** Boiss. et A. Huet	Gelincik, Lale	Petals	Cough	Waited in water 10 days	Int	0.31	(1)^b^	—
Papaveraceae, MARE 21930								
***Papaver rhoeas*** L.	Gelincik, Lale	Petals	Cough	Waited in water 10 days	Int	0.52	(6, 7)^b^	Cough (26, 27, 31)
Papaveraceae, MARE 21725								(22, 25, 26, 27, 28, 29, 34, 35, 36, 39, 44, 45, 48,51)^b^
***Phillyrea latifolia*** L.	Akcakesme, Kesme	Leaves	**Kidney stones**	Infusion	Int	0.19	—	—
Oleaceae, MARE 21067, 21134, 21295, 21507								
***Pinus brutia*** Ten.Pinaceae, MARE 20155, 21556	Cam		Cough	Crushed with sugar	Eaten before breakfast, int	0.63	Cough (1, 7)	—
		Young shoots Resin	Stomach ulcer	Decoction	Before breakfast for 40 days, int		Shortness of breath (3, 9)	
			Shortness of breath	—	Eaten 1 teaspoon, int		Ulcer (9, 10)	
		Immature cones	Cough	Boiled with milk	Ext		(1, 2, 3, 5, 6, 7, 9, 10, 11, 15)^b^	
			Diaper rush (babies)	Heated then mixed with olive oil	Ext		—	
		Immature cones	Shortness of breath	Decoction	Int		—	
		Terebinthine	Fracture	—	Ext		—	
***Pistacia terebinthus*** L. subsp. ***palaestina*** (Boiss.) Engler	Menegic	Fruits	Cough	Roasted the boiled with water (made coffee)	Int	0.27	(3, 5, 6)^b^	—
Anacardiaceae, MARE 21005, 20101, 21057, 21159, 21176		Latex	Wounds	—	Ext			
***Plantago lanceolata*** L. Plantaginaceae, MARE 21877	Kirksinir otu, Pisikuyrugu	Leaves	Rheumatism, wounds, abscess	Crushed	Wrapped in a cloth, wait for	0.71	Wounds (3, 6, 8)	Abscess (25, 27)
							(3, 5, 8)^b^	Rheumatism (50)
							—	Wounds (24, 25, 26, 44, 53), (13, 16, 17, 22, 24, 25, 26, 27, 28, 29, 32, 33, 34, 35, 36, 39, 41, 44, 48, 50, 54)^b^
***Plantago major*** L. subsp. ***intermedia*** (Gilib.) lange	Kirksinir otu	Leaves	Rheumatism, wounds	Crushed	Wrapped in a cloth, wait for 10 min, ext	0.71	Wounds (3, 9)	—
Plantaginaceae, MARE 20184							(3, 9, 10)^b^	
***Polygonum cognatum*** Meisn.	Madimak	Aerial parts	**Kidney stones**	Infusion	Int	0.10	—	—
Polygonaceae, MARE 21923, 21939								
***Primula vulgaris*** Huds. subsp. ***vulgaris***	Ezrail meneksesi, Sari menekse	Flowers	Cold	Infusion	Int	0.09	—	(16, 31, 50)^b^
Primulaceae, MARE 21697 [*Primula vulgaris* Huds.]								
***Punica granatum*** L.^a^	Nar	Seeds	Diarrhea	Boiled for 2–3 h	Int	0.52	Diarrhea (3,6,7,8)	Diarrhea (22.54)
Lythraceae, MARE 21659		Exocarp	Diarrhea	Infusion	Int		(6.8)^b^	(17,, 22, 28, 35, 48)^b^
***Quercus coccifera*** L. Fagaceae, MARE 21008, 21074	Kotoprini, Piynar	Fruits, roots	Enuresis (in child)	Decoction	Int	0.77	Burns (1,3,9)	(12, 41, 52)^b^
			Wounds	Decoction (added olive oil)	Ext		Wounds (9)	
			Burns	Decoction	Int		(3,4,6)^b^	
			Menstrual pain	Decoction	Ext		—	
***Rhus coriaria*** L. Anacardiaceae, MARE 20171, 21041, 21055, 21100, 21128	Sumak	Fruits	Foot odor	—	Put in socks, ext	0.72	Foot odor (9)	(15, 51)^b^
		Leaves and fruits	Tinea pedis	Bath	Ext		Gingivitis (9)	
		Leaves	Gingivitis	Infusion	Gargle		Toothache (10)	
		Aerial parts	Pelade	Infusion	Ext		(4,6,9,10.11)^b^	
		—	Skin diseases	Decoction	Ext		—	
		—	Eczema	Decoction	Ext		—	
		—	Toothache	Decoction	Gargle		—	
***Rosa canina*** L.	Itburnu, Kusburnu	Fruits	Diabetes	Decoction	Int	0.16	Diabetes (6)	Diabetes (15)
Rosaceae, MARE 21911							(6, 8, 9, 10)^b^	(12, 13, 15, 16, 17, 22, 23, 27, 28, 31, 34, 35, 37, 38, 39, 41, 44, 46, 51, 52, 53, 54)^b^
***Rosmarinus officinalis*** L.^a^	Arizmari, Biberiye	Leaves	Stomach diseases	Infusion	Int	0.38	(1, 4, 7, 9, 10, 11)^b^	Stomach diseases (54)
Lamiaceae, MARE 20083, 20148, 21069, 21131, 21338								(12, 13, 15, 16, 17, 19, 20, 21, 22, 24, 25, 27, 28, 29, 31, 35, 36, 40, 41, 43, 44, 48, 50, 51, 52, 53, 54)^b^
***Rubus sanctus*** Schreb. Rosaceae, MARE 21555	Bogurtlen	Roots, leaves	Kidney ailments	Decoction	Int	0.44	Diabetes (1.8)	Diabetes (1, 8), (15, 41)^b^
			Diabetes, Emenagog	Infusion	Gargle		Kidney ailments (9)	
			Sore throat	Crushed	Ext		Menstrual pains (9)	
			Burns	—	—		(1, 3, 6, 7, 8, 9, 11)^b^	
***Rumex*** sp.	Kalmik cayi, Cerkez cayi	Aerial parts	Cardiovascular system diseases	Boiled in milk	Int	0.24	—	—
Polygonaceae, MARE 21668 ^a^								
***Ruscus aculeatus*** L. var. ***aculeatus***	Kandak	Fruits	Haemorrhoids	Infusion	Int	0.39	Kidney stones (6)	(13, 22, 27, 28, 51)^b^
Asparagaceae, MARE 20100 [*Ruscus aculeatus* L.]		Roots	Kidney stones	Decoction	Int			
***Salvia fruticosa*** Mill.	Adacayi, Faskomila	Aerial parts	Cold	Infusion	Int	0.53	Cold (7)	(15)^b^
Lamiaceae, MARE 20092							(4, 5, 7)^b^	
***Salvia viridis*** L.	Esek cayi	Aerial parts	Stomachache	Infusion	Int	0.37	Stomachache (6)	—
Lamiaceae, MARE 21668, 21710, 21712								
***Sambucus nigra*** L.	Kokarot, Bandirik	Fruits	Haemorrhoids	—	Int	0.13	(8, 9, 10)^b^	Hemorrhoids (17, 25, 27, 29)
Adoxaceae, MARE 21002								(13, 15, 16, 17, 20, 21, 24, 25, 26, 27, 28, 29, 31, 32, 33, 34, 35, 36, 37, 38, 39, 41, 42, 43, 44, 45, 47, 48, 49, 50, 51, 53, 54,55)^b^
***Sideritis cilicica*** Boiss. et Balansa	Dag cayi	Aerial parts	**Cold**	Infusion	Int	0.61	—	
Lamiaceae, MARE 21552								
***Sideritis congesta*** P. H. Davis et Hub.-Mor	Dag cayi	Arial parts	Cold, Abdominal pain	Infusion	Int	0.61	(4, 11)^b^	—
Lamiaceae, MARE 20101a, 21,520								
***Solanum nigrum*** L. subsp. ***schultesii*** (Opiz) Wessely	Boncuklu gogundurme	Leaves	Wounds	Boiled in water added flour	Ext	0.29	(8)^b^	(36)^b^
Solanaceae, MARE 20186 [*Solanum decipiens* opiz]								
***Stachys lavandulifolia*** vahl var. ***lavandulifolia***	Tuylu cay	Aerial parts	Cold	Infusion	Int	0.17	Cold (9.10)	—
Lamiaceae, MARE 21933 [*Stachys lavandulifolia* vahl]							(5,9,10,11)^b^	
***Tamarix smyrnensis*** Bunge	Ilgin	Stem bark	Antipyretic	Decoction	Int	0.31	(3)^b^	—
Tamaricaeae, MARE 21146								
***Teucrium polium*** L.	Aci yavsan	Aerial parts	Abdominal pain	Infusion	Int	0.65	Abdominal pain (3)	Stomachache (38)
Lamiaceae, MARE 20090, 20196, 21011, 21018			Stomachache	Crushed with olive oil added flour, wrapped in a cloth, infusion	Ext., Int		Stomachache (1, 5, 10, 11), (1, 3, 4, 5, 6, 9, 10, 11)^b^	(38, 50)^b^
***Tilia rubra*** DC. subsp. ***caucasica*** (Rupr.) V. Engl	Ihlamur	Flowers	Cold	Decoction	Int	0.31	Common cold (1)	—
Malvaceae, MARE 20098			Cough	Infusion	Int			
***Tribulus terrestris*** L.	Devecokerten, demirpitirak, Ucdis	Aerial parts	Cardiovascular system diseases, kidney ailments	Decoction	Int	0.44	(3, 4, 6, 8, 9, 11)^b^	(15)^b^
Zygophyllaceae, MARE 20059, 21024, 21136			Enuresis	Infusion	Int			
***Urginea maritima*** (L.) Baker	Zipcik sogani, zibgin	Bulbus	Rheumatism	Sliced	Wrapped in a cloth, ext	0.37	Rheumatism (4,9,10)	(17, 22, 27, 40, 51)^b^
Asparagaceae, MARE 21089 [*Drimia maritima* (L.) Stearn]							(9, 10)^b^	
***Urtica urens*** L.	Isirgan	Leaves	Rheumatism	Crushed	Wrapped in a cloth, ext	0.61	Rheumatism (4)	Diuretic (17)
Urticaceae, MARE 21297, 21510		Aerial parts	Diuretic	Infusion	Int		(1, 4, 9)^b^	Rheumatism (17, 22, 36, 44) (12, 14, 17, 19, 22, 27, 28, 35, 36, 44, 48, 51)^b^
***Verbascum*** sp.	Paskulak, Sigirkulagi, Salkaba	Flowers	Stomachache	Infusion	Int	0.62	(1, 9, 10)^b^	(38, 50)^b^
Scrophulariaceae, MARE 21026			Constipation	Decoction	Int			
***Viscum album*** L. subsp. ***abietis*** (Wiesb.) Abromeit	Govelek	Whole plants	Cardiovascular system diseases	Decoction	Int	0.36	(6, 9, 10)^b^	—
Santalaceae, MARE 21006, 21081								
***Viscum*** album L. subsp. ***album***	Govelek	Whole plants	Cardiovascular system diseases	Infusion	Int	0.36	(3, 4, 5)^b^	Heart problems (19)
Santalaceae, MARE 21027, 21147 [*Viscum album* L.]								(12, 13, 19, 24, 43, 48, 50, 54)^b^
***Vitex agnus-castus*** L.	Hayit	Fruits	Cardiovascular system diseases	Infusion	Int	0.17	(1, 5, 7, 9, 10)^b^	(42)^b^
Lamiaceae, MARE 21073, 21124, 21179		Leaves	Fertility (in women)	Infusion	Int			
***Xanthium strumarium*** L. subsp. ***cavanillesii*** (Schouw) D. Löve et P. Dansereau	Pitirak	Aerial parts	Wounds	Crushed	Wrapped in a cloth, ext. Wrapped in a cloth, ext	0.40	—	—
Asteraceae, MARE 21083 [*Xanthium orientale* subsp. *italicum* (Moretti) Greuter]		Leaves	Abscess	Crushed				

Int.; Internal use. Ext.; External use. Adm.: Administration.

a Cultivated plant.

b Different usage, the new plant uses were marked as bold.

(1) Akaydin et al., 2013, (2) Ari et al., 2018, (3) Bulut et al., 2017, (4) Everest and Ozturk, 2005, (5) Fakir et al., 2016, (6) Gunes et al., 2017, (7) Gurdal and Kultur, 2013, (8) Guzel et al., 2015, (9) Sargin, 2015, (10) Sargin and Buyukcengiz, 2019, (11) Yesilada et al., 1993, (12) Agelet and Vallès, 2003, (13) Akerreta et al., 2007a, (14) Akerreta et al., 2007b, (15) Axiotis et al., 2018, (16) Bellia and Pieroni, 20,015, (17) Benítez et al., 2010, (18) Benítez et al., 2012, (19) Calvo and Cavero, 2014, (20) Calvo et al., 2011, (21) Camejo-Rodrigues et al., 2003, (22) Carrió and Vallès, 2012, (23) Cavero et al., 2011a, (24) Cavero et al., 2011b, (25) Cornara et al., 2009, (26) De Natale and Pollio, 2007, (27) González et al., 2010, (28) Gras et al., 2019, (29) Guarrera et al., 2005, (30) Łuczaj et al., 2019, (31) Matejic et al., 2020, (32) Mattalia et al., 2020a, (33) Mattalia et al., 2020b, (34) Mattalia et al., 2020c, (35) Mautone et al., 2019, (36) Menale and Muoio, 2014, (37) Mustafa et al., 2012, (38) Mustafa et al., 2015, (39) Mustafa et al., 2020, (40) Novais et al., 2004, (41) Papageorgiou et al., 2020, (42) Parada et al., 2009, (43) Petrakou et al., 2020, (44) Pieroni, 2000, (45) Pieroni et al., 2002, (46) Pieroni, 2017, (47) Rigat et al., 2007, (48) Rigat et al., 2013, (49) Sansanelli et al., 2017, (50) Sari´c-Kundali´c et al., 2010, (51) Tuttolomondo et al., 2014, (52) Viegi, et al., 2003, (53)Vinagre et al., 2019, (54) Vitasović Kosić et al., 2017, (55) Zivkovic et al., 2020.

**TABLE 2:** The plants used in veterinary medicine in Mersin (Turkey).

Botanical name, family and specimen number	Local name	Plant part used	Ailments treated/Therapeutic effect	Preparation	Administration	UV	Similar usage in literature
***Euphorbia helioscopia*** L.	Sutlegen	Latex	Warts, wounds	—	Ext	0.41	Wounds (52)
Euphorbiaceae, MARE 21300			Snakebite (for goat)	—	Int		
***Glaucium flavum*** Crantz	Okuzbogurden	Aerail parts	Emetic	—	Int	0.07	—
Papaveraceae, MARE 21870							
***Juniperus foetidissima*** Willd.	Ardic, Yayli ardic	Aerial parts	Antiseptic (after birth)	—	Ext	0.13	—
Cupressaceae, MARE 21061, 21506, 21924							
***Hypericum perforatum*** L.	Kantaron	Aerial parts	Wounds	Oleat	Ext	0.47	Wounds (29.52)
Hypericaceae, MARE 20080, 20997, 21091, 21549							
***Mentha longifolia*** (L.) Hudson subsp. ***typhoides*** (Briq.) Harley var. **t*yphoides***	Yarpiz	Aerial parts	Anthelmintic	Infusion	Int	0.24	—
Lamiaceae, MARE 21016, 21125 [*Mentha longifolia* subsp. *typhoides* (Briq.) Harley]							
***Nerium oleander*** L.	Agu, Sindilag, Zakkum	Flowers	Scabies	Decoction	Ext	0.31	(52)^b^
Apocynaceae, MARE 20166, 21088, 21130							
***Punica granatum*** L.^a^	Nar	Exocarp	Diarrhea	Infusion	Int	0.31	—
Lythraceae, MARE 21659							
***Rhus coriaria*** L.	Sumak	Young shoots	Diarrhea	—	Int	0.50	—
Anacardiaceae, MARE 20171, 21041, 21055, 21100, 21128							
***Salix alba*** L.	Sogut	Young shoots	Diarrhea	—	Int	0.09	(52)^b^
Salicaceae, MARE 20137							

Int.; Internal use. Ext.; External use.

a Cultivated plant.

b Different usage; the new plant uses were marked as bold.

The UV data is summarized in the statistical data analysis section. Amongst the most commonly used plants were *Hypericum* species. During our interviews, participants shared that they learned about using the oleate of *Hypericum* species for external wound treatment from their ancestors, emphasizing that it was even used for sword wounds in ancient times. We even observed that many of the participants’ kept this oleate in their homes.

The fruit of *Arum dioscoridis* Sm, is the leading herb used in the treatment of haemorrhoids in the region. The leaves are boiled and consumed as food while fruits are used as toys.

We recorded that the latex of *Euphorbia helioscopia* L., E. *kotschyana* Fenzl*, E. rigida* M. Bieb.*, Glaucium flavum* Crantz and *Ficus carica* L. are used for the treatment of warts in the region. *F. carica* latex is also used for toothaches.

Molasses “pekmez” prepared from the fruits of *C. siliqua* and *J. drupaceae*, which are very common in the flora of the region, was traditional product used in children and adults, especially in upper respiratory tract diseases, and was also sold in the local markets.

Female participants over 60 years of age, who contributed to our research in the region, mentioned that the roots of *Malva* species were previously used to terminate pregnancies when birth control methods were not common, and that their mothers frequently applied this method.

*Helichrysum compactum* Boiss., *S. cilicica* and *S. congesta* P. H. Davis et Hub.-Mor. are endemic species of the region with therapeutic usages (presented in Table 1). *S. cilicica* and *S. congesta* were the most consumed herbal teas in the region and are cultivated in the gardens of some participants.

*Gundelia tournefortii* L. var*, tournefortii* and *Pistacia terebinthus* L. subsp. *palaestina* (Boiss.) Engler were used to prepare a special traditional coffee. In addition, fruits of *P. terebinthus* were used as a snack and sold in local bazaars.

*M.communis* is used in treatments for diabetes and constipation*,* and its fruits are also consumed as a snack. Another application we recorded in almost every village in our study was its usage during cemetery visits.

*Euphorbia helioscopia* L., *Glaucium flavum* Crantz, *J. foetidissima*, *H. perforatum*, *N. oleander*, *Mentha longifolia* (L.) Hudson subsp. *typhoides* (Briq.) Harley var. *typhoides*, *Punica granatum* L. and *Rhus coriaria* L. are used in the treatment of both humans and animals. Among the medicinal plants used for veterinary purposes, we found that only *Salix alba* L. is used exclusively for the treatment of animals (Table 2).

#### Plant Parts Used and Methods of Preparation

The parts of plants used for medicinal purposes were aerial parts (26.8%), leaves (18.4%), fruits (15.1%) and flowers (7%). The main preparation methods using these parts were infusion (27.6%), direct application (22.2% without any preparation procedure), decoction (18.9%), application after crushing (11.4%), and other less common methods (19.9%).

A total of 189 drugs were recorded in this study. Most were used internally (55.7%) (Table 1, Table 2). Olive oil, flour, honey and sugar were used as additional ingredients in the preparation of these remedies.

The medicinal plants used in multiherbal recipes containing two or more species are presented in Table 3. A decoction prepared from *R. coriaria* and *Q. coccifera* is used in the treatment of warts and a mixture prepared from *P. brutia* and *H. perforatum* is used in stomach disorders.

**TABLE 3:** Multiherbal recipes used as folk medicine in Mersin.

**R**ecipe	Plant	Plant part used	Ailments treated, therapeutic effect	Preparation	Administration
1	***Rhus coriaria***	Leaves	Warts Decoction Ext.	Stomach ailments crushed and mixed with oleat	Before breakfast 1 × 1, int.
	***Quercus coccifera***	Roots		
	***Pinus brutia***	Resin		
	***Hypericum perforatum***	Aerial parts		

#### Plant Names

Local names of medicinal plants are also recorded in this study. The names of the all plants in Turkish, as well as some Cretan plant names, were recorded during the study. Some of these plants have vernacular names that are also used for different plant species, potentially leading to complications. These are presented in Table 4, where we see that in some cases different species of the same genus have the same common names.

**TABLE 4:** The same vernacular name was used for more than one plant species.

Local name	Botanical names, family and specimen numbers			
Aysefatma	***Alcea pallida*** (Willd.) Waldst. et Kit.	***Alcea rosea*** L.	—	—
	Malvaceae, MARE 20130, 21063	Malvaceae, MARE 20173		
Sutlegen	***Euphorbia helioscopia*** L	***Euphorbia kotschyana*** Fenzl	***Euphorbia rigida*** M. Bieb	—
	Euphorbiaceae, MARE 21300	Euphorbiaceae, MARE 21092	Euphorbiaceae, MARE 21318	
Kantaron	***Hypericum atomarium*** Boiss	***Hypericum lydium*** Boiss.	***Hypericum montbretii*** Spach	***Hypericum perforatum*** L.
	Hypericaceae, MARE 20117	Hypericaceae, MARE 21097	Hypericaceae, MARE 20067	Hypericaceae, MARE 20080, 20997, 21091, 21549
Ardic	***Juniperus foetidissima*** willd	***Juniperus oxycedrus*** L. subsp. ***oxycedrus***	***Juniperus excelsa*** M. Bieb.	—
	Cupressaceae, MARE 21061, 21506, 21924	Cupressaceae, MARE 20160, 21103, 21505 (*Juniperus oxycedrus* L.)	Cupressaceae, MARE 20193, 21003, 21059	
Ebegumeci	***Malva parviflora*** L.	***Malva sylvestris*** L	—	—
	Malvaceae, MARE 20088, 21034	Malvaceae, MARE 21951		
Gomec	***Malva parviflora*** L.	***Malva sylvestris*** L.	—	—
	Malvaceae, MARE 20088, 21034	Malvaceae, MARE 21951		
Kekik	***Origanum syriacum*** L. var. ***bevanii*** (Holmes) Ietswaart	***Origanum vulgare*** L.	—	—
	Lamiaceae, MARE 20078, 20082, 20103, 21166, 21330 [*Origanum syriacum* subsp. *bevanii* (Holmes) Greuter et Burdet]	Lamiaceae, MARE 21020		
Kirksinir otu	***Plantago lanceolata*** L	***Plantago major*** L. subsp. ***intermedia*** (Gilib.) lange	—	—
	Plantaginaceae, MARE 21877	Plantaginaceae, MARE 20184		
Dag cayi	***Sideritis cilicica*** Boiss. et Balansa	***Sideritis congesta*** P. H. Davis et Hub.-Mor.	—	—
	Lamiaceae, MARE 21552	Lamiaceae, MARE 20101a, 21,520		
Govelek	***Viscum album*** L. subsp. ***abietis*** (wiesb.) Abromeit	***Viscum*** album L. subsp. ***album***	—	—
	Loranthaceae, MARE 21006, 21081	Santalaceae, MARE 21027, 21147 (*Viscum album* L.)		

#### Statistical Data Analysis

**FIGURE 9:**
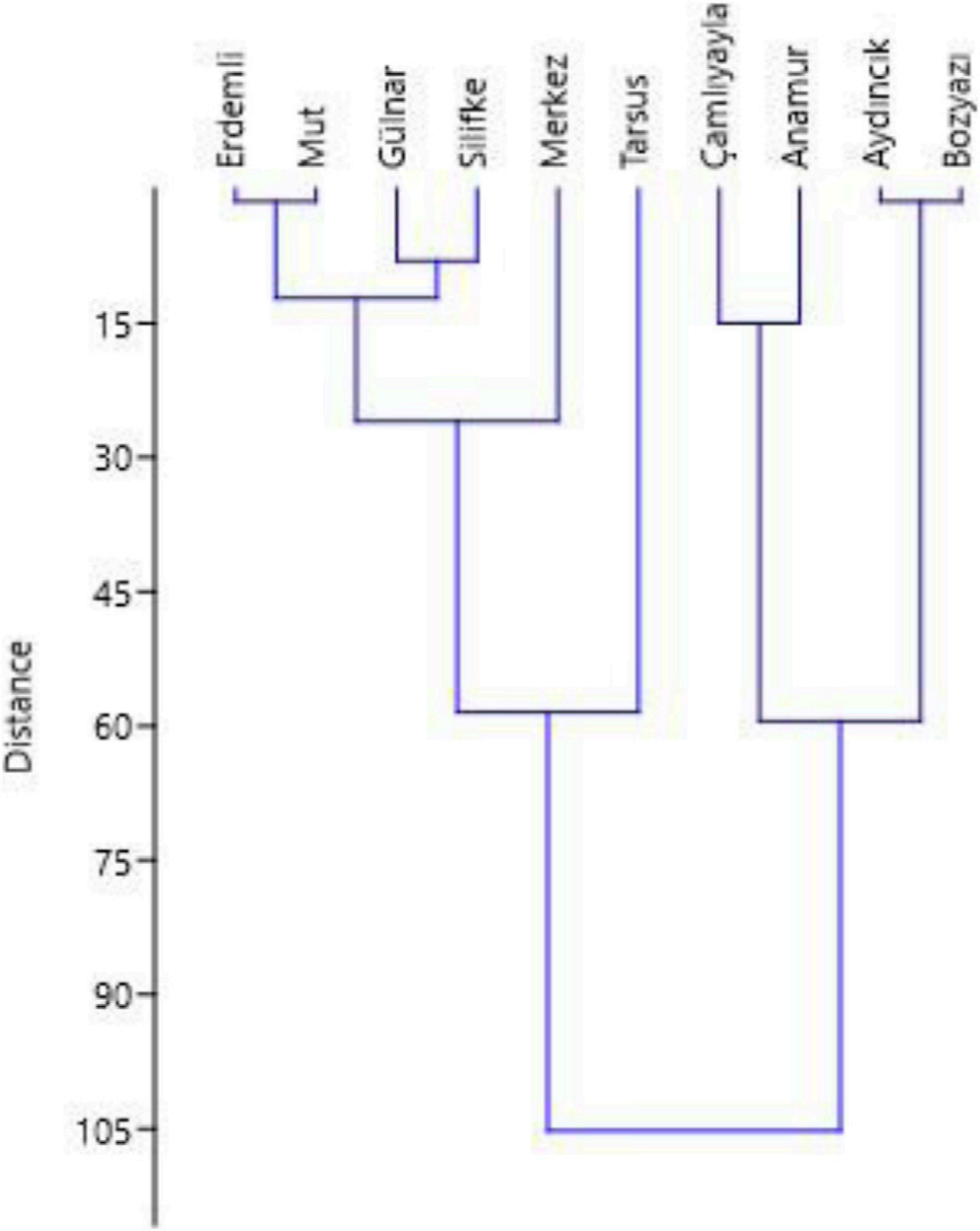
Dendogram showing UPGMA clustering (with Euclide distance) of districts (those where over 10 interviews were carried out).

Analysis of the diversity and similarity among districts, based on the ten districts, using species abundance and amount of information on treatment usage, was carried out by hierarchical clustering (Figure 9). The analysis resulted in five main clusters at the truncation point of 20. Erdemli, Mut, Gulnar and Silifke, which are close to one another, showed greater similarity among themselves. Similarly, Aydincik and Bozyazi, which are proximate to one another, also displayed very similar characteristics. Interestingly, there was a close similarity between Anamur and Camliyayla, despite them being far apart. Merkez and Tarsus were both different from the other districts, but Tarsus was the most distinct among the districts.

The proportion test was used to compare the true (population) proportion of the population who recognize and use these species in the various districts. The proportions are given below: 1-Camliyayla (0.72), 2-Tarsus (0.82), 3-Merkez (0.88), 4-Mut (0.66), 5-Anamur (0.60), 6-Silifke (0.91), 7-Gülnar (0.92) and 8-Erdemli (0.86). Bozyazı and Aydıncık districts wasn’t included.

The *p*-value was 0.0005773 < 0.05. We conclude that there is a significant difference between the districts in terms of awareness of the species. The pairwise comparison with Holm adjustment was conducted to detect the differences between the districts. The difference between 5–3 (*p* value = 0.044) and 5–6 (*p* value = 0.042) are significant. This result indicates that the major source of difference was the district (Anamur). We can interpret this to mean that Anamur uses fewer species in the traditional treatments than the other districts.

The proportion test was also used to compare the true (population) UV index for the species. As a result of our analysis, the plants with the highest UV values are *H. perforatum* (0.80), *C. libani* (0.78), *Q. coccifera* (0.77), *Arum dioscoridis* (0.76) and J. drupaceae (0.74), which are presented in Table 1.

After analysis, the *p*-value is obtained as 0.4423 > 0.05. It is concluded that there is no significant difference between the five most commonly used species in terms of UV.

According to the FIC results, gastrointestinal system diseases (mainly stomach ailments) had the highest value at 0.77, followed by skin and subcutaneous tissues (mainly wound healing) at 0.72, circulatory system (mainly haemorrhoids) at 0.60, respiratory system (mainly cold) at 0.57, urinary/genital system (0.41), musculo-skeletal (mainly rheumatism, 0.44) and finally metabolism (mainly diabetes, 0.35) disorders (Table 5).

**TABLE 5:** FIC values of category of ailments.

Ailment categories	Informant consensus factor (FIC)
Gastrointestinal system	0.77
Skin and subcutaneous tissues	0.72
Circulatory system	0.64
Respiratory system	0.57
Bones, joints, etc.	0.44
Genito-urinary system	0.41
Metabolism	0.35
Infectious diseases	0.29
Sensory organs	0.14
Veterinary uses	0.51

### Folk Remedies and Related Knowledge Originating From Animals

This research determines that some animals, which constitute an important part of biological diversity, are used for medical purposes in addition to plants used as traditional folk medicine in Mersin. Because animal-based folk remedies are a part of traditional therapy, we present them in this study alongside plants. The folk remedies derived from animals (*n* = 110) recorded during fieldwork *via* interviews with informants are presented in Table 6.

**TABLE 6:** Animal-based folk remedies used in Mersin for treating human diseases.

Remedy	Preparation and administration	Ailments treated/Therapeutic effect	Similar usage in literature
Bee’s wax	Ext	Bruises	(1)^a^
Camel milk	Boiled, per oral, int	Cough	—
Catfish	Externally applied	Fracture	—
Chicken tail	Ext	Abortive	—
Donkey’s milk	Per oral, int	Cough (in children)	1
Donkey urine	Ext	Scorpion sting	(1)^a^
Jay	Consumed, int	Speech delay	—
Porcupine	Consumed, int	Haemorrhoids	—
Pork	Consumed, int	Cracked heels	—
Rabbit lard	Dropped into the ear, ext	Earache	—
Snail shell	Ext	Wounds	—
Snake skin	Applied on scalp, ext	Headache	—
	Applied on skin, ext	Acne	
Talpa	Consumed, int	Goiter	—

1 Pieroni et al., 2011.

a Different usage.

We observed that local people dealing with animal husbandry and hunting as a hobby in the area reaching from villages near the coast to the slopes of the Taurus Mountains were more knowledgeable in this regard.

We found that the use of hedgehog meat for haemorrhoid treatment is very common in the region. The participants added that it is very tasty alongside its therapeutic properties. In addition to the use of animals or animal products for human health, it is very common to use tortoize shell against the evil eye, especially among Yoruks. Furthermore, women and young girls of the village were said to knit with hair from the tails of horses when they could not find thread in Camliyayla, where needle lace is a common traditional handicraft. For this reason, the owners of white horses have to keep their horses tied up in their barns.

We were also informed that the calabash (*Lagenaria* sp.), known as “Kaplankabak” in the Gülnar area, is used as an instrument to make sound that keeps predators away to protect people living in tents. A piece of tanned goat skin is stretched across the calabash and a rope is inserted into a hole in the skin. An intense noise is produced when the rope is pulled (Supplementary Video S1).

## Discussion

### Comparison With Previous Studies

Comprehensive ethnobotanical studies previously carried out in neighboring areas (Yeşilada et al., 1993; Everest and Ozturk, 2005; Akaydın et al., 2013; Arı et al., 2018; Gürdal and Kültür, 2013; Güzel et al., 2015; Sargin, 2015; Fakir et al., 2016; Bulut et al., 2017; Güneş et al., 2017; Sargin and Büyükcengiz, 2019) found that *P. brutia* was the most commonly used herbal medicinal plant at ten localities in Mersin and its environs. Our findings compared with previous studies can be seen in Table 1, Table 2.

In previous studies, widely distributed species *A. cilicica, C. libani, C. siliqua, H. perforatum, J. drupaceae, J. oxycedrus, L. nobilis, M. communis and O. syriacum* subsp. *bevanii* were found to be the major plants used in traditional folk medicines. The most commonly used method for preparation in Mersin is infusion (Akaydın et al., 2013; Sargin, 2015; Sargın et al., 2015; Sargin and Büyükcengiz, 2019).

Sargın et al., 2015; Sargin, 2015; Sargin and Büyükcengiz, 2019 noted that the fruits of *C. siliqua* and *J. drupaceae* in particular were used for “molasses” in the region. In addition, *L. nobilis,* locally known as “teynel,” is commonly used for medicinal purposes. Its leaves are used as a spice and during summer in the process of drying fruits to be eaten in winter. The plant is also commonly used in herbal soaps and sold in local markets. Our results agree with these previous findings.

Sargın et al., 2015; Sargin, 2015, Sargin and Büyükcengiz, 2019 collected species belonging to the genera *Dactylorhiza*, *Ophrys*, *Orchis* and *Serapias* and noted that they were used in salep and ice cream production. Unfortunately, we were not able to collect these plants, although we also received information on their usage. We are able to contribute information not recorded previously on the widely cultivated plant *Citrus lemon* L. (Osbeck), which is used to make ice cream in Kaleburcu village under the leadership of the mukhtar.

Besides corroborating previous data in our study, we record new 36 plant taxa with medicinal usages in Mersin (Table 1). Furthermore, nine plants used for applications in animal health were also recorded in this study for the first time in this region.

Plants that have been recorded in previous ethnobotanical studies in Turkey and other Balkan and Mediterranean countries are also presented in Table 1, Table 2 (Agelet and Vallès, 2003; Akerreta et al., 2007a; Akerreta et al., 2007b; Axiotis et al., 2018; Bellia and Pieroni, 2015; Benítez et al., 2010; Benítez et al., 2012; Calvo and Cavero, 2014; Calvo et al., 2011; Camejo-Rodrigues et al., 2003; Carrió and Vallès, 2012; Cavero et al., 2011a; Cavero et al., 2011b; Cornara et al., 2009; De Natale and Pollio, 2007; González et al., 2010; Gras et al., 2019; Guarrera et al., 2005; Łuczaj et al., 2019; Matejic et al., 2020; Mattalia et al., 2020a; Mattalia et al., 2020b; Mattalia et al., 2020c; Mautone et al., 2019; Menale and Muoio, 2014; Mustafa et al., 2012; Mustafa et al., 2015; Mustafa et al., 2020; Novais et al., 2004, Papageorgiou et al., 2020; Parada et al., 2009; Petrakou et al., 2020; Pieroni, 2000; Pieroni et al., 2002; Pieroni, 2017; Rigat et al., 2007; Rigat et al., 2013; Sansanelli et al., 2017; Sari´c-Kundali´c et al., 2010; Tuttolomondo et al., 2014; Vinagre et al., 2019; Vitasović Kosić et al., 2017; Zivkovic et al., 2020) The medicinal uses of the species in Mersin were compatible with previous findings; such as *Asphodelus aestivus* Brot. (wounds), *Cydonia oblonga* Mill. (diarrhea and stomach-ache), *Ecballium elaterium* (L.). A. Rich. (sinusitis), *Equisetum telmateia* Ehrh. (diuretic), *Ficus carica* L. (wart), *Foeniculum vulgare* Mill. (abdominal pain), *H. perforatum* (wound and haemorrhoids), *Juglans regia* L. (haemorrhoids)*, L. nobilis* (rheumatism), *M. sylvestris* L. (abdominal pain), *Mentha* x *piperita* L. (abdominal pain), *P. lanceolata* L. (wounds), *Punica granatum* (diarrhea), *Rosa canina* L. (diabetes) and *Urtica urens* L. (rheumatism). Further studies on some of the listed species support the folkloric uses of these plants with new evidence: the wound-healing properties of *P. brutia* have been shown (Cetin et al., 2013); *H. perforatum* has been predominantly used for treating depression, wounds and ulcers (EMA 2006 https://www.ema.europa); *Rosa canina* has been studied for antidiabetic properties (Rahimi et al., 2020); and the antihypertensive, anti-inflammatory and anti-ulcerogenic properties of *Cydonia oblonga* have been investigated (Zhouet al., 2014).

To the best of our knowledge; usages of *Arbutus* x *andrachnoides* Link, *Asphodeline lutea* (L.) Rchb.*, Asphodelus fistulous* L., *Cistus salviifolius* L.*, G. flavum*, *H. compactum, H. atomarium* Boiss.*, H. lydium* Boiss.*, Phillyrea latifolia*, *Polygonum cognatum* Meisn. and *S. cilicica* have been recorded for the first time in the region, these new applications are indicated in bold in Table 1; however, of the folk medicinal plants with veterinary uses (Table 2), *Euphorbia helioscopia, H. perforatum, N. oleander* and *S. alba* have also been listed in other studies in the field (Guarrera et al., 2005; Viegi et al., 2003).

### Harmful Effects of Medicinal Plants

The harmful effects of the medicinal plants were also discussed during the interviews. The informants stated that *E. elaterium* and *Drimia maritima* (L.) Stearn should be used carefully due to side effects and contra-indications.

In addition, we recorded that the fruits of *Atropa belladonna* L., a medicinal plant that is not used medicinally in the region, were eaten by T. G. (age 23), who had seen his father eat this plant to quench his thirst in Inkoyu. T.G. said that he ate many fruits of this plant while he was traveling with his cousin, but his cousin only tasted it. He noted that he experienced poisoning (hallucinations, dry mouth, poor vision) in the hours after consuming the fruit and that he had to go to a hospital far from the village.

### Review of Local Plant Names

In our research, we also found some local plant names not recorded in other studies (Akaydın et al., 2013; Sargin, 2015; Sargin and Büyükcengiz, 2019). These are: Akcakesme, Altinbas, Aysefatma, Ayigulu, Boncuklu Gogundurme, Esefatma cicegi, Ezrail meneksesi, Gomec, Hasbi cicegi, Ilgin Kandak, Karahan, Kecirisi, Kesme, Kuskonmaz, Madimak, Pisikuyrugu, Sari menekse, Selvi, and Yosun cayi. Some vernacular names of the medicinal plants recorded for the first time in Turkey in this study include Boncuklu gogundurme, Esefatma cicegi, Ezrail meneksesi and Yosun cayi (Tuzlacı, 2011).

Although Turkish is spoken in all settlements in the region, some villages were established after migration events. For example, a village of Circassian immigrants has preserved their language and the elders speak Circassian among themselves. There is also a village formed by Cretan immigrants after the population exchange with Greece. The villagers speak Cretan among themselves, which allowed us to record the Cretan names of some plants during our research. Greek plant names are recorded and transcribed in the Latin alphabet. Some of these names were included in a study conducted on the island of Lemnos (Papageorgiou et al., 2020).

### Review of Traditional Healing With Animals

Comparing our limited data on zootherapy in the Balkans and the Mediterranean region with the study of Pieroni et al., 2011; we find that the use of donkey milk against cough is common, unlike the use of donkey urine and beeswax. Though limited, we believe these data will contribute to future studies to be conducted by the experts in this field.

### Quantitative Findings

Comparing our UV values with those of other studies conducted in Mersin, we see that while *C. libani* was 0.64 in our study, Sargın et al., 2015 recorded a value of 0.36 for this plant. Our value for *Q. coccifera* was 0.77 UV, but 0.50 in the study of Akaydın et al., 2013. Another high UV value in our study was 0.87 for *T. polium* (0.71), which was determined to be 0.57 and 0.35, respectively, in previous studies (Akaydın et al., 2013; Sargin and Büyükcengiz, 2019). *H. perforatum*, which has the highest value in our study, was calculated as 0.53 in Sargın, 2015 and 0.42 in Akaydın et al., 2013.

FIC (ICF) values have also been investigated for Mersin in previous studies and were found to be highest for analgesics (0.78), cardiovascular diseases (0.76) and kidney problems (0.70) (Sargın et al., 2015); while another study had the highest value (0.74) for haemorrhoids, followed by gastrointestinal diseases, nutrition disorders, obesity (0.53) and cardiovascular diseases (0.51) (Sargın 2015). In a recent study the highest FIC value calculated was for the treatment category of livestock diseases (0.78), followed by analgesics (0.67) and kidney problems (0.62) (Sargin and Büyükcengiz, 2019). In another study carried out in Mersin (Akaydın et al., 2013), the FIC ratios recorded were: respiratory system (0.88), gastrointestinal diseases (0.79), dermatological disorders (0.76) and urinary disorders (0.69).

The most common usages of the plants we found were for stomach disorders, wound, haemorrhoids and colds, although the previous studies found different rates of usage (Akaydın et al., 2013; Sargin et al., 2015; Sargın 2015; Sargin and Büyükcengiz, 2019).

## Conclusion

In this comprehensive ethnobotanical study which for the first time evaluates the entire province, we find that 93 folk medicinal plants belonging to 43 families are still being used in Mersin. Among these, the medicinal usages of 36 taxa are new records in Mersin. The usage of nine taxa in veterinary medicine are also recorded. People living in rural areas who could also benefit from modern facilities and technology, maintain a connection, which we regard as a cultural bridge in this study, to traditional knowledge in their daily lives. We also note that the younger generation living in villages more freely shared their knowledge while participating in the study. Although we can say that the transfer of traditional knowledge continues despite visual and electronic culture and the increased use of technology reaching even the most remote villages, we must also note that if this research had been carried out a few decades ago, it would have obtained more comprehensive results in terms of the use of traditional knowledge based on botanical diversity. A connection between the old and young generations that is key to the preservation of important knowledge was established during the interviews. Performing ethnobotanical studies could revitalize this bridge of knowledge between old and new generations and help form solid foundation for its preservation. As a conclusion, study also demonstrates that a historically and culturally important province with a rich flora such as Mersin has great potential as a source of traditional ethnobotanical knowledge.

## Data Availability

The raw data supporting the conclusion of this article will be made available by the authors, without undue reservation, to any qualified researcher.

## APPENDIX 1: Questionnaire Form

1 Name and surname of the participant.
2 Age and sex of the participant.
3 Telephone and address of the participant.
4 Educational level of the participant.
5 Date of interview.
6 Place of residence of the participant.
7 Duration of residence of the participant.
8 Local name of the plant.
9 Human health or Animal health.
10 Ailments treated/therapeutic effect.
11 Plant part used.
12 Preparation.
13 Administration.
14 Dosage.
15 Duration of treatment.
16 Age group of patients (baby, child, adult).
17 Side effects.
18 Different ethnobotanical use.
19 Animal based remedies.

## References

[B1] Abu-Irmaileh B. E. Afifi F. U. (2003). Herbal Medicine in Jordan with Special Emphasis on Commonly Used Herbs. J. Ethnopharmacology 89, 193–197. 10.1016/s0378-8741(03)00283-6 14611882

[B2] Afifi F. U. Abu-Irmaileh B. (2000). Herbal Medicine in Jordan with Special Emphasis on Less Commonly Used Medicinal Herbs. J. Ethnopharmacology 72, 101–110. 10.1016/s0378-8741(00)00215-4 10967460

[B3] Agelet A. Vallès J. (2003). Studies on Pharmaceutical Ethnobotany in the Region of Pallars (Pyrenees, Catalonia, Iberian Peninsula). Part II. New or Very Rare Uses of Previously Known Medicinal Plants. J. Ethnopharmacology 84, 211–227. 10.1016/S0378-8741(02)00319-7 12648818

[B4] Ahmet Sargin S. (2015). Ethnobotanical Survey of Medicinal Plants in Bozyazı District of Mersin, Turkey. J. Ethnopharmacology 173, 105–126. 10.1016/j.jep.2015.07.009 26190351

[B5] Akaydin G. Şimşek I. Arituluk Z. C. Yeşilada E. (2013). An Ethnobotanical Survey in Selected Towns of the Mediterranean Subregion (Turkey). Turkish J. Biol. 37, 230–247. 10.3906/biy-1010-139

[B6] Akerreta S. Cavero R. Y. Calvo M. I. (2007a). First Comprehensive Contribution to Medical Ethnobotany of Western Pyrenees. J. Ethnobiol. Ethnomedicine 3. 10.1186/1746-4269-3-26 PMC1904192 17553138

[B7] Akerreta S. Cavero R. Y. López V. Calvo M. I. (2007b). Analyzing Factors that Influence the Folk Use and Phytonomy of 18 Medicinal Plants in Navarra. J. Ethnobiol. Ethnomedicine 3, 1–18. 10.1186/1746-4269-3-16 PMC1868015 17433105

[B8] Axiotis E. Halabalaki M. Skaltsounis L. A. (2018). An Ethnobotanical Study of Medicinal Plants in the Greek Islands of North Aegean Region. Front. Pharmacol. 9, 1–6. 10.3389/fphar.2018.00409 29875656 PMC5974156

[B9] Bailey K. (1994). Typologies and Taxonomies: An Intriduction to Classification Techniques. London-New Delhi: Sage Publications. 10.4135/9781412986397

[B10] Bellia G. Pieroni A. (2015). Isolated, but Transnational: The Glocal Nature of Waldensian Ethnobotany, Western Alps, NW Italy. J. Ethnobiol. Ethnomedicine 11. 10.1186/s13002-015-0027-1 PMC4495842 25948116

[B11] Benítez G. González-Tejero M. R. Molero-Mesa J. (2010). Pharmaceutical Ethnobotany in the Western Part of Granada Province (Southern Spain): Ethnopharmacological Synthesis. J. Ethnopharmacology 129, 87–105. 10.1016/j.jep.2010.02.016 20226847

[B12] Benítez G. González-Tejero M. R. Molero-Mesa J. (2012). Knowledge of Ethnoveterinary Medicine in the Province of Granada, Andalusia, Spain. J. Ethnopharmacology 139, 429–439. 10.1016/j.jep.2011.11.029 22155471

[B13] Bulut G. Tuzlaci E. (2013). An Ethnobotanical Study of Medicinal Plants in Turgutlu (Manisa-Turkey). J. Ethnopharmacology 149, 633–647. 10.1016/j.jep.2013.07.016 23933313

[B14] Bulut G. Haznedaroğlu M. Z. Doğan A. Koyu H. Tuzlacı E. (2017). An Ethnobotanical Study of Medicinal Plants in Acipayam (Denizli-Turkey). J. Herbal Med. 10, 64–81. 10.1016/j.hermed.2017.08.001

[B15] Calvo M. I. Cavero R. Y. (2014). Medicinal Plants Used for Cardiovascular Diseases in Navarra and Their Validation from Official Sources. J. Ethnopharmacology 157, 268–273. 10.1016/j.jep.2014.09.047 25304200

[B16] Calvo M. I. Akerreta S. Cavero R. Y. (2011). Pharmaceutical Ethnobotany in the Riverside of Navarra (Iberian Peninsula). J. Ethnopharmacology 135, 22–33. 10.1016/j.jep.2011.02.016 21345364

[B17] Camejo-Rodrigues J. Ascensão L. Bonet M. À. Vallès J. (2003). An ethnobotanical study of medicinal and aromatic plants in the Natural Park of "Serra de São Mamede" (Portugal). J. Ethnopharmacology 89, 199–209. 10.1016/S0378-8741(03)00270-8 14611883

[B18] Carrió E. Vallès J. (2012). Ethnobotany of Medicinal Plants Used in Eastern Mallorca (Balearic Islands, Mediterranean Sea). J. Ethnopharmacology 141, 1021–1040. 10.1016/j.jep.2012.03.049 22783553

[B19] Cavero R. Y. Akerreta S. Calvo M. I. (2011a). Pharmaceutical Ethnobotany in Northern Navarra (Iberian Peninsula). J. Ethnopharmacology 133, 138–146. 10.1016/j.jep.2010.09.019 20883764

[B20] Cavero R. Y. Akerreta S. Calvo M. I. (2011b). Pharmaceutical Ethnobotany in the Middle Navarra (Iberian Peninsula). J. Ethnopharmacology 137, 844–855. 10.1016/j.jep.2011.07.001 21767624

[B21] Cornara L. La Rocca A. Marsili S. Mariotti M. G. (2009). Traditional Uses of Plants in the Eastern Riviera (Liguria, Italy). J. Ethnopharmacology 125, 16–30. 10.1016/j.jep.2009.06.021 19563876

[B22] Davis P. H. Mill R. R. Tan K. (1988). Flora of Turkey and the East Aegean Islands. (Edinburgh: Edinburgh University Press), 10.

[B23] Davis P. H. (Eds) (1965). Flora of Turkey and the East Aegean Islands. (Edinburgh: Edinburgh University Press), 1–9.

[B24] De Natale A. Pollio A. (2007). Plants Species in the Folk Medicine of Montecorvino Rovella (Inland Campania, Italy). J. Ethnopharmacology 109, 295–303. 10.1016/j.jep.2006.07.038 16987626

[B25] EMA (2020). European Medicines Agency (EMA). Available at: (https://www.ema.europea.eu (Accessed December 25, 2020).

[B26] Everest A. Ozturk E. (2005). Focusing on the Ethnobotanical Uses of Plants in Mersin and Adana Provinces (Turkey). J. Ethnobiol. Ethnomedicine 1, 6. 10.1186/1746-4269-1-6 PMC1277086 16270936

[B28] Fakir H. Korkmaz M. Icel B. (2016). Medicinal Plants Traditionally Used for Pain Alleviation in Antalya Province, Turkey. Stud. Ethno-Medicine 10, 314–324. 10.1080/09735070.2016.11905503

[B29] Meteroloji Genel Müdürlüğü (2020). WMO 2020 Küresel İklim Durumu Nihai Raporu. Available at: (http://www.mgm.gov.trAccessed October 29, 2020).

[B30] González J. A. García-Barriuso M. Amich F. (2010). Ethnobotanical study of medicinal plants traditionally used in the Arribes del Duero, western Spain. J. Ethnopharmacology 131, 343–355. 10.1016/j.jep.2010.07.022 20643201

[B31] Gras A. Serrasolses G. Vallès J. Garnatje T. (2019). Traditional Knowledge in Semi-rural Close to Industrial Areas: Ethnobotanical Studies in Western Gironès (Catalonia, Iberian Peninsula). J. Ethnobiol. Ethnomedicine 15, 1–37. 10.1186/s13002-019-0295-2 PMC6444684 30940210

[B32] Guarrera P. M. Forti G. Marignoli S. (2005). Ethnobotanical and Ethnomedicinal Uses of Plants in the District of Acquapendente (Latium, Central Italy). J. Ethnopharmacology 96, 429–444. 10.1016/j.jep.2004.09.014 15619562

[B33] Guner A. Ozhatay N. Ekim T. Baser K. H. C. (2000). The Flora of Turkey and the East Aegean Islands, 11. Edinburgh: Edinburgh University Press.

[B34] Guner A. Kandemir A. Menemen Y. Yıldırım H. Aslan S. Ekşi G. (2018). Illustrated Flora of Turkey, 2. İstanbul: ANG Vakfı Nezahat Gökyiğit Botanik Bahçesi Yayınları (in Turkish).

[B35] Güneş S. Savran A. Paksoy M. Y. Koşar M. Çakılcıoğlu U. (2017). Ethnopharmacological Survey of Medicinal Plants in Karaisalı and its Surrounding (Adana-Turkey). J. Herbal Med. 8, 68–75. 10.1016/j.hermed.2017.04.002

[B36] Gürdal B. Kültür Ş. (2013). An Ethnobotanical Study of Medicinal Plants in Marmaris (Muğla, Turkey). J. Ethnopharmacology 146, 113–126. 10.1016/j.jep.2012.12.012 23261486

[B37] Güzel Y. Güzelşemme M. Miski M. (2015). Ethnobotany of Medicinal Plants Used in Antakya: A Multicultural District in Hatay Province of Turkey. J. Ethnopharmacology 174, 118–152. 10.1016/j.jep.2015.07.042 26239155

[B38] Heinrich M. Ankli A. Frei B. Weimann C. Sticher O. (1998). Medicinal Plants in Mexico: Healers' Consensus and Cultural Importance. Soc. Sci. Med. 47, 1859–1871. 10.1016/s0277-9536(98)00181-6 9877354

[B39] Heinrich M. Lardos A. Leonti M. Weckerle C. Willcox M. Applequist W. (2018). Best Practice in Research: Consensus Statement on Ethnopharmacological Field Studies - ConSEFS. J. Ethnopharmacology 211, 329–339. 10.1016/j.jep.2017.08.015 28818646

[B40] International Society of Ethnobiology (ISE) (2008). International Society of Ethnobiology Code of Ethics. Available at: (https://www.ethnobiology.net/what-wedo/core-programs/ise-ethics-program/code-of-ethics/Accessed October 25, 2020).

[B41] Łuczaj Ł. Jug-Dujaković M. Dolina K. Jeričević M. Vitasović-Kosić I. (2019). The Ethnobotany and Biogeography of Wild Vegetables in the Adriatic Islands. J. Ethnobiol. Ethnomedicine 15, 1–17. 10.1186/s13002-019-0297-0 PMC6440087 30922334

[B43] Matejić J. S. Stefanović N. Ivković M. Živanović N. Marin P. D. Džamić A. M. (2020). Traditional Uses of Autochthonous Medicinal and Ritual Plants and Other Remedies for Health in Eastern and South-Eastern Serbia. J. Ethnopharmacol. 261, 113186. 10.1016/j.jep.2020.113186 32730888

[B44] Mattalia G. Sõukand R. Corvo P. (2020a). The Virtues of Being Peripheral, Recreational, and Transnational, Transnational: Local Wild Food and Medicinal Plant Knowledge in Selected Remote Municipalities of Calabria, Southern Italy. Ethnobot. Res. Appl. 19, 1–20. 10.32859/era.19.38.1-20

[B45] Mattalia G. Sõukand R. Corvo P. Pieroni A. (2020b). Blended Divergences: Local Food and Medicinal Plant Uses Among Arbëreshë, Occitans, and Autochthonous Calabrians Living in Calabria, Southern Italy. Plant Biosyst. 154, 615–626. 10.1080/11263504.2019.1651786

[B46] Mattalia G. Sõukand R. Corvo P. Pieroni A. (2020c). Dissymmetry at the Border: Wild Food and Medicinal Ethnobotany of Slovenes and Friulians in NE Italy. Econ. Bot. 74, 1–14. 10.1007/s12231020-09488-y

[B47] Mautone M. De Martino L. De Feo V. (2019). Ethnobotanical research in Cava de' Tirreni area, Southern Italy. J. Ethnobiol. Ethnomedicine 15, 1–21. 10.1186/s13002-019-0330-3 PMC6798482 31623655

[B48] Menale B. Muoio R. (2014). Use of Medicinal Plants in the South-Eastern Area of the Partenio Regional Park (Campania, Southern Italy). J. Ethnopharmacol. 153, 297–307. 10.1016/j.jep.2014.02.039 24583106

[B49] Mustafa B. Hajdari A. Krasniqi F. Hoxha E. Ademi H. Quave C. L. (2012). Medical Ethnobotany of the Albanian Alps in Kosovo. J. Ethnobiol. Ethnomed. 8, 1–14. 10.1186/1746-4269-8-6 22284581 PMC3285519

[B50] Mustafa B. Hajdari A. Pieroni A. Pulaj B. Koro X. Quave C. L. (2015). A Cross-Cultural Comparison of Folk Plant Uses Among Albanians, Bosniaks, Gorani and Turks Living in South Kosovo. J*.* Ethnobiol. Ethnomed. 11, 1–26. 10.1186/s13002-015-0023-5 25964167 PMC4449527

[B51] Mustafa B. Hajdari A. Pulaj B. Quave C. L. Pieroni A. (2020). Medical and Food Ethnobotany Among Albanians and Serbs Living in the Shtërpcë/Štrpce Area, South Kosovo. J. Herb. Med. 22, 100344. 10.1016/j.hermed.2020.100344

[B52] Novais M. H. Santos I. Mendes S. Pinto-Gomes C. (2004). Studies on Pharmaceutical Ethnobotany in Arrabida Natural Park (Portugal). J. Ethnopharmacol. 93, 183–195. 10.1016/j.jep.2004.02.015 15234752

[B53] Papageorgiou D. Bebeli P. J. Panitsa M. Schunko C. (2020). Local Knowledge about Sustainable Harvesting and Availability of Wild Medicinal Plant Species in Lemnos Island, Greece. *J. Ethnobiol.* Ethnomed 16, 1–23. 10.1186/s13002-020-00390-4 32560660 PMC7304145

[B54] Parada M. Carrió E. Bonet M. À. Vallès J. (2009). Ethnobotany of the Alt Empordà Region (Catalonia, Iberian Peninsula). Plants Used in Human Traditional Medicine. J. Ethnopharmacol. 124, 609618. 10.1016/j.jep.2009.04.050 19422899

[B55] Petrakou K. Iatrou G. Lamari F. N. (2020). Ethnopharmacological Survey of Medicinal Plants Traded in Herbal Markets in the Peloponnisos, Greece. J. Herb. Med. 19, 100305. 10.1016/j.hermed.2019.100305

[B56] Phillips O. Gentry A. H. (1993). The Useful Plants of Tambopata, Peru: II. Additional Hypothesis Testing in Quantitative Ethnobotany. Econ. Bot. 47, 33–43. 10.1007/BF02862204

[B57] Pieroni A. Quave C. Nebel S. Heinrich M. (2002). Ethnopharmacy of the Ethnic Albanians (Arbëreshë) of Northern Basilicata, Italy. Fitoterapia 73, 217–241. 10.1016/s0367-326x(02)00063-1 12048017

[B58] Pieroni A. Giusti M. E. Quave C. L. (2011). Cross-Cultural Ethnobiology in the Western Balkans: Medical Ethnobotany and Ethnozoology Among Albanians and Serbs in the Pešter Plateau, Sandžak, South-Western Serbia. Hum. Ecol. 39, 333–349. 10.1007/s10745-011-9401-3

[B59] Pieroni A. (2000). Medicinal Plants and Food Medicines in the Folk Traditions of the Upper Lucca Province, Italy. J. Ethnopharmacology 70, 235–273. 10.1016/S0378-8741(99)00207-X 10837988

[B60] Pieroni A. (2017). Traditional Uses of Wild Food Plants, Medicinal Plants, and Domestic Remedies in Albanian, Aromanian and Macedonian Villages in South-Eastern Albania. J. Herbal Med. 9, 81–90. 10.1016/j.hermed.2017.05.001

[B61] Rahimi M. Sajadimajd S. Mahdian Z. Hemmati M. Malekkhatabi P. Bahrami G. (2020). Characterization and Anti-diabetic Effects of the Oligosaccharide Fraction Isolated from Rosa Canina in STZ-Induced Diabetic Rats. Carbohydr. Res. 489, 107927. 10.1016/j.carres.2020.107927 32062396

[B62] Rigat M. Bonet M. À. Garcia S. Garnatje T. Vallès J. (2007). Studies on Pharmaceutical Ethnobotany in the High River Ter valley (Pyrenees, Catalonia, Iberian Peninsula). J. Ethnopharmacology 113, 267–277. 10.1016/j.jep.2007.06.004 17656056

[B63] Rigat M. Vallès J. Iglésias J. Garnatje T. (2013). Traditional and Alternative Natural Therapeutic Products Used in the Treatment of Respiratory Tract Infectious Diseases in the Eastern Catalan Pyrenees (Iberian Peninsula). J. Ethnopharmacology 148, 411–422. 10.1016/j.jep.2013.04.022 23612419

[B64] Sansanelli S. Ferri M. Salinitro M. Tassoni A. (2017). Ethnobotanical Survey of Wild Food Plants Traditionally Collected and Consumed in the Middle Agri Valley (Basilicata Region, Southern Italy). J. Ethnobiol. Ethnomedicine 13, 50. 10.1186/s13002-017-0177-4 PMC5586000 28874202

[B65] Sargin S. A. Büyükcengiz M. (2019). Plants Used in Ethnomedicinal Practices in Gulnar District of Mersin, Turkey. J. Herbal Med. 15, 100224. 10.1016/j.hermed.2018.06.003

[B66] Sargin S. A. Selvi S. Büyükcengiz M. (2015). Ethnomedicinal Plants of Aydıncık District of Mersin, Turkey. J. Ethnopharmacology 174, 200–216. 10.1016/j.jep.2015.08.008 26278812

[B67] Sargin S. A. (2015). Ethnobotanical Survey of Medicinal Plants in Bozyazı District of Mersin, Turkey. J. Ethnopharmacol. 173, 105–126. 10.1016/j.jep.2015.07.009 26190351

[B68] Šarić-Kundalić B. Dobeš C. Klatte-Asselmeyer V. Saukel J. (2010). Ethnobotanical Study on Medicinal Use of Wild and Cultivated Plants in Middle, South and West Bosnia and Herzegovina. J. Ethnopharmacol. 131, 33–55. 10.1016/j.jep.2010.05.061 20594943

[B69] Scherrer A. M. Motti R. Weckerle C. S. (2005). Traditional Plant Use in the Areas of Monte Vesole and Ascea, Cilento National Park (Campania, Southern Italy). J. Ethnopharmacology 97, 129–143. 10.1016/j.jep.2004.11.002 15652287

[B70] Sokal R. Michener C. (1958). A Statistical Method for Evaluating Systematic Relationships. University of Kansas Science Bulletin, 38, 1409–1438.

[B72] Teklehaymanot T. Giday M. (2007). Ethnobotanical Study of Medicinal Plants Used by People in Zegie Peninsula, Northwestern Ethiopia. J. Ethnobiol. Ethnomed. 3, 12. 10.1186/1746-4269-3-12 17355645 PMC1852296

[B73] The Plant List (2013). A Working List of All Plant Species. Available at: http://www.theplantlist.org (Accessed October 15, 2020).

[B27] Thomas E. Vandebroek I. Sanca S. Van Damme P. (2009). Cultural Significance of Medicinal Plant Families and Species Among Quechua Farmers in Apillapampa, Bolivia. J. Ethnopharmacology 122 (1), 60–67. 10.1016/j.jep.2008.11.021 19101618

[B74] Trotter R. T. Logan M. H. (1986). “Informant Consensus: a New Approach for Identifying Potentially Effective Medicinal Plants,” in Plants in Indigenous Medicine and Diet. Editor Etkin N. L. (Bedford Hills, New York: Redgrave Publishing Company), 91–112.

[B75] TUIK (2020). Turkiye Istatistik Kurumu (TUIK) Türkiye Istatik Kurumu. Available at: https://www.tuik.gov.tr (Accessed October 28, 2020).

[B76] Tuttolomondo T. Licata M. Leto C. Savo V. Bonsangue G. Letizia Gargano M. (2014). Ethnobotanical Investigation on Wild Medicinal Plants in the Monti Sicani Regional Park (Sicily, Italy). J. Ethnopharmacology 153, 568–586. 10.1016/j.jep.2014.02.032 24632020

[B77] Tuzlacı E. (2011). *Türkiye Bitkileri Sözlüğü “A Dictionary Of Turkish Plants*”. second ed. İstanbul: Alfa Yayınları.

[B79] Varga F. Šolić I. Jug Dujaković M. Łuczaj Ł. Grdiša M. (2019). The First Contribution to the Ethnobotany of Inland Dalmatia: Medicinal and Wild Food Plants of the Knin Area, Croatia. Acta Soc. Bot. Pol. 88 (2), 3622. 10.5586/asbp.3622

[B80] Vinagre C. Vinagre S. Carrilho E. (2019). The use of medicinal plants by the population from the Protected Landscape of “serra de Montejunto”, Portugal. J. Ethnobiol. Ethnomedicine 15, 1–30. 10.1186/s13002-019-0309-0 PMC6604383 31262314

[B81] Vitasović Kosić I. Juračak J. Łuczaj Ł. (2017). Using Ellenberg-Pignatti Values to Estimate Habitat Preferences of Wild Food and Medicinal Plants: An Example from Northeastern Istria (Croatia). J. Ethnobiol. Ethnomed. 13, 2-19. 10.1186/s13002-017-0159-6 28577572 PMC5457627

[B82] Yeşilada E. Honda G. Sezik E. Tabata M. Goto K. Ikeshiro Y. (1993). Traditional Medicine in Turkey. IV. Folk Medicine in the Mediterranean Subdivision. J. Ethnopharmacol. 39, 31–38. 10.1016/0378-8741(93)90048-a 8331960

[B83] Zhou W.-t. Abdurahman A. Abdusalam E. Yiming W. Abliz P. Aji Q. (2014). Effect of *Cydonia Oblonga* Mill. Leaf Extracts or Captopril on Blood Pressure and Related Biomarkers in Renal Hypertensive Rats. J. Ethnopharmacology 153, 635–640. 10.1016/j.jep.2014.03.014 24661965

[B84] Živković J. Ilić M. Šavikin K. Zdunić G. Ilić A. Stojković D. (2020). Traditional Use of Medicinal Plants in South-Eastern Serbia (Pčinja District): Ethnopharmacological Investigation on the Current Status and Comparison with Half a Century Old Data. Front. Pharmacol. 11, 1–12. 10.3389/fphar.2020.01020 32733251 PMC7360817

